# ’More of the same, but worse than before’: A qualitative study of the challenges encountered by people who use drugs in Nova Scotia, Canada during COVID-19

**DOI:** 10.1371/journal.pone.0283979

**Published:** 2023-04-05

**Authors:** Emilie Comeau, Matthew Bonn, Sheila Wildeman, Matthew Herder

**Affiliations:** 1 Faculty of Medicine, Dalhousie University, Halifax, Nova Scotia, Canada; 2 Canadian Association of People Who Use Drugs, Dartmouth, Nova Scotia, Canada; 3 Health Law Institute, Schulich School of Law, Dalhousie University, Halifax, Nova Scotia, Canada; Universitat Luzern, SWITZERLAND

## Abstract

**Background:**

To learn about the experiences of people who use drugs, specifically opioids, in the Halifax Regional Municipality (HRM), in Nova Scotia, Canada during the COVID-19 pandemic through qualitative interviews with people who use drugs and healthcare providers (HCP). This study took place within the HRM, a municipality of 448,500 people [1]. During the pandemic many critical services were interrupted while overdose events increased. We wanted to understand the experiences of people who use drugs as well as their HCPs during the first year of the pandemic.

**Methodology:**

We conducted a qualitative study using semi-structured interviews with 13 people who use drugs and 6 HCPs, including physicians who work in addiction medicine (3), a pharmacist, a nurse, and a community-based opioid agonist therapy (OAT) program staff member. Participants were recruited within HRM. Interviews were held via phone or videoconference due to social distancing directives. Interviews focused on the challenges people who use drugs and HCPs faced during the pandemic as well as elicited perspectives on a safe supply of drugs and the associated barriers and facilitators to the provision of a safe supply.

**Results:**

Of the 13 people who use drugs who participated in this study, ages ranged from 21–55 years (mean 40). Individuals had spent on average 17 years in HRM. Most people who use drugs (85%, n = 11) utilized income assistance, the Canadian Emergency Response Benefit, or disability support. Many had experienced homelessness (85%, n = 11) and almost half (46%, n = 6) were currently precariously housed in the shelter system. The main themes among interviews (people who use drugs and HCPs) were housing, accessing healthcare and community services, shifts in the drug supply, and perspectives on safe supply.

**Conclusions:**

We identified several challenges that people who use drugs face in general, but especially during the COVID-19 pandemic. Access to services, housing support, and interventions to use safely at home were limited. As many challenges faced by people who use drugs exist outside of COVID-19, we concluded that the formal and informal interventions and changes in practice that were made to support people who use drugs should be sustained well past the end of the pandemic. The need for enhanced community supports and a safe supply of drugs, despite its complicated nature, is essential for the health and safety of people who use drugs in HRM, especially during COVID-19.

## Background

The overdose death epidemic has been impacting the lives of Canadians long before the global COVID-19 pandemic. Between January 2016 and June 2021 there were over 32, 632 opioid toxicity deaths in Canada alone, averaging over 20 a day [2]. Overdosing on opioids can cause respiratory depression and possibly death via unopposed stimulation of opioid receptors. Ingestion of excessive amounts of opioids usually occurs unintentionally, especially with fentanyl (a much stronger opioid) contaminating street opioid supplies. During the first year of the pandemic, there was a 96% increase in opioid-related deaths and the number of deaths has continued to remain high during the COVID-19 pandemic [3]. In short, there have been three waves to the overdose epidemic: at first the mortality was driven by the malpractice of pharmaceutical marketing practices; then by street sourced opioids such as heroin; and now it is driven by powerful novel synthetic opioids, particularly fentanyl, and fentanyl analogues, which have contaminated the unregulated (street) drug market [4]. Arguably, there is a new wave to the overdose epidemic: the syndemic of HIV, Hepatitis C Virus (HCV), homelessness, overdose, and COVID-19 [5]. Public health directives to stay home and adhere to social distancing guidelines created unique challenges for people who use drugs as most must either visit a pharmacy daily to access opioid agonist therapy (OAT) or rely on a dangerous unregulated drug market. The illegal drug supply became particularly unstable as drug trafficking routes had to change due to international travel shutting down and borders closing during the pandemic [6].

In response to this syndemic, the Canadian government has started to recognize not only the health and safety risks faced by people who use drugs, but also the stigma and discrimination they encounter, particularly people who must attend the pharmacy daily to receive OAT medications. Coupled with witnessed urine drug screen (UDS) analyses, the requirement to physically attend pharmacies every day is inconvenient, stigmatizing, and dehumanizing [7,8]. During the first wave of COVID-19, Health Canada took a progressive upstream approach by creating an exemption to the Canadian *Controlled Drugs and Substances Act* (CDSA), allowing people who use drugs to stay home to self-isolate [9]. This exemption gave pharmacists the flexibility to renew, extend, and transfer narcotic prescriptions; permitted practitioners to verbally prescribe prescriptions of controlled substances; and, permitted individuals to deliver controlled substances to patients. As a result, people who use drugs were supported in maintaining access to prescribed drugs, including narcotics. However, given the increase in opioid-related deaths during the first several months of the pandemic, early measures such as these proved insufficient. Ontario saw increasing rates of opioid-related deaths in the first 6 months of the COVID-19 pandemic, with 1237 deaths between March and September 2020 [10]. In British Columbia, a record number of opioid-related deaths was reported during this time and they are now reporting 210 suspected illicit drug toxicity deaths in December 2022 alone (11)

Another harm reduction approach that has gained traction in recent years is based in the concept of a “safe supply” of drugs, although the precise meaning of safe supply and how it is to be operationalized are the subject of debate [11]. The federal government, for instance, is supportive of safe supply provided it occurs through an established health care provider (HCP)-patient relationship. Rather than foreclosing other options for safe supply (e.g. compassion clubs, which historically provide a safe space for people to access medicines and connect with a range of health services) [12], we understand the concept according to the broad and inclusive definition adopted by Canadian Association of People Who Use Drugs (CAPUD). In this view, safe supply is simply “a legal and regulated supply of drugs with mind-body altering properties that were traditionally offered illegally” [13,14]. In comparison to safe supply, *safer* supply is a “harm reduction driven, public health approach that involves the provision of a pharmaceutical drug supply of known quantity and quality to adults who use illegal drugs…” but as per activist groups, does not adequately address the expressed needs of people who use drugs. An example of safer supply is a medicalized model involving regulated prescribing of injectable OAT (iOAT) [15]. Advocates have been arguing for a domestic safe supply of diacetylmorphine (i.e., heroin), which is supported by a large international body of peer reviewed scientific literature [14,16–18], yet still only have very few clinics in Canada (Vancouver and Victoria, British Columbia) provide access to this main opioid people are seeking.

In a qualitative study, Foreman-Mackey and colleagues (2022) describe safe supply from the perspective of professional stakeholders such as program managers, executive directors, health authority representatives, and healthcare providers. Acknowledging that safe supply is not a “one-size-fits-all” approach, they define safe supply as a “low-barrier access to substances of known quality and quantity, offered on a continuum from prescribed to a legal, regulated supply, and focused on upholding autonomy and liberation of people who use drugs” [19]. Other researchers are looking to people who use drugs to define safe(r) supply, such as with concept mapping, in order to inform programs moving forward [20]. Kilmer and Pardo (2022) further emphasize the ambiguity of “safe supply” and encourage its clarification in order to enrich policy discussions. They fear that conflating prescription models of safe supply with other approaches may create barriers to piloting and evaluating new interventions [21].

Proponents of safe supply would argue that it saves lives by decreasing overdose events due to an unpredictable, unregulated drug market, however multiple barriers to prescribing, such as restrictive laws and fear of discipline from regulatory colleges, have been elicited in previous studies [22]. Critics of safe supply most often highlight the risk of diversion, which can be described as any non-intended or non-medical use of a prescribed medication, or use by any individual other than for whom it was prescribed. Another concern among critics is that providing substances legally could perpetuate substance use and undermine treatment options such as OAT [23]. Of note, safe supply can be distinguished from OAT (methadone, buprenorphine, slow-release oral morphine) by their overarching goals. Often the goal with OAT is to facilitate abstinence from drugs whereas safe supply refers to providing a safer alternative to the toxic illegal drug supply for people at high risk of overdose [24].

In Nova Scotia, where this study took place, the government has not yet indicated support for safe supply; however, the provincial health authority offered one-time bridge funding for ReFIX–the only overdose prevention site (OPS) currently operating in Nova Scotia [25]. ReFIX replaced Atlantic Canada’s first Urgent Public Health Need Site, “HaliFIX,” which was opened and operated without provincial support [26]. In 2021, the Nova Scotia Health Authority pledged $500,000 to support two new OPS sites over a two year period, which led to the opening of ReFIX and a community based organization in Cape Breton, in the northern part of the province.

The present qualitative study describes the experiences of people who use drugs, specifically opioids and their HCPs during the first wave of the COVID-19 pandemic in HRM. As we will show, people who use drugs faced numerous serious health, social, and economic challenges in accessing essential services during the pandemic. Harm reduction services shut down (or reduced capacity), shelters and food banks closed, and many jobs were lost—all while people who use drugs were trying to access an increasingly unstable supply of adulterated opioids and other drugs [27]. Therefore, given the risks involved in either obtaining drugs or withdrawing from them suddenly, our primary research aim was to understand what, if any, government and/or community-led interventions assisted people who use drugs during the start of the pandemic. A secondary aim was to elicit people who use drugs’ and HCPs conceptions of a safe supply of drugs as well as potential or real barriers and facilitators to realizing safe supply in the local context. Given the time of our study, the relevant context necessarily included COVID-19. We were also interested in exploring what, if any, models of safe supply (formal or informal) were offered in HRM during COVID-19 (physician-led, user-led, or other). We explain how these findings can inform person-centered strategies to improve access to quality healthcare and a safe supply of drugs, both in and beyond HRM.

## Methodology

This qualitative study was conducted through Dalhousie Medical School as part of its Research in Medicine Program. Ms Comeau and Mr Herder had full access to all of the data in the study and take responsibility for the integrity of the data and the accuracy of the data analysis. The principal investigator Emilie Comeau (EC) was responsible for interviewing (via zoom, telephone conversations), transcribing interviews, and drafting the manuscript. Matthew Bonn (MB) supported EC in recruitment. MB and Matthew Herder (MH) were involved with the conceptual design, analysis, and drafting of the manuscript. Sheila Wildeman (SW) reviewed the manuscript and provided edits, comments, and suggestions to strengthen the manuscript. MH supervised and obtained funding for this project. All authors contributed equally to critical revision of the manuscript for important intellectual content, although only EC and MH had full access to the data (de-identified interview transcript excerpts surrounding the included quotations can be found under Supplementary Information).

### Participants

People eligible for recruitment were adults (>18 years of age) living in HRM who spoke English. Recruitment was restricted to HRM to understand the limitations and successes in this local context. The sample size (n = 19) was determined based on maximizing the number of interviews performed to obtain saturation of themes while also working within the constraints of providing appropriate honorariums to each participant. The majority of participants were people who use drugs, primarily opioids (13). We also recruited HCPs (6) to gain an understanding of their first-hand perspectives, although our focus was on the perspectives of people who use drugs. Inclusion criteria for HCPs were quite broad: they had to work in HRM and provide healthcare services to people who use drugs; professional titles were self-reported. Recruitment of people who use drugs occurred with support from CAPUD, who advertised the study via their social media pages (Facebook, Twitter, Instagram). CAPUD staff also agreed to use word of mouth for recruiting people who use drugs with whom they had previously established a trusting relationship. We also relied on ‘snowball sampling’ from participants by their telling others about the research project and sharing our contact information. Recruitment for HCPs was referred by MB and MH via email, then followed up via email by the PI.

Of the 13 people who use drugs who participated, ages ranged from 21–55 years (mean = 40). During the time of the interviews, almost all (85%, n = 11) people who use drugs were receiving income assistance, the Canada Emergency Response Benefit (CERB) distributed by the federal government, or disability support, except for one individual who had only recently been released from prison and another who did not disclose their income status. Individuals had spent a range of 2 months to 55 years in HRM (average 17 years). People experiencing homelessness were overrepresented, as 11 individuals (85%, n = 11) currently or previously experienced homelessness. Almost half (46%, n = 6) were precariously housed in the shelter system, two of whom were housed in hotels during the pandemic. Of those not in the shelter system, one was staying with a partner, one was with a stranger and two were staying with family members. Three (23%, n = 3) were living in their own apartment. The most cited reason for participating in this research project was a desire to educate the public about drug use during the pandemic (38%, n = 5), with monetary reimbursement as the second most cited answer (23%, n = 3). No information was gathered on gender, race or ethnicity (although some participants self-identified their gender). The HCPs included three physicians (2 attending staff, 1 resident physician), a pharmacist, a nurse, and a community-based OAT staff member. All of the HCPs worked primarily with people who use drugs.

### Data collection and analysis

Ethical approval for this study was granted by Dalhousie University’s Research Ethics Board (REB File #: 2020–5149). All participants gave verbal informed consent and agreed to being quoted, provided they were not identifiable. All participants (people who use drugs and HCPs) were offered a $25 honorarium for their time and expertise. Interviews were conducted by the PI over the phone or via secure videoconferencing software, due to social distancing directives. The interviews lasted between 19–90 minutes (mean = 40 minutes) and focused on exploring people who use drugs’ experiences during the pandemic. Special focus was placed on which services helped or hindered people who use drugs during the pandemic, as well as their conceptions of safe supply. Interviews with HCP’s concentrated on their experiences providing care to people who use drugs during the pandemic. All interviews were done using a semi-structured open-ended interview guide (see Supplemental Information for sample). Only interviews with people who use drugs included demographic questions such as housing, income, and drug use history as we were focused on centering the voices of people who use drugs, complimented by the perspectives of their HCPs. As such, we did not investigate HCP demographics in this study. As interviews progressed, the interview guide was adjusted to reflect pertinent issues that came up in prior interviews. For example, issues related to housing and experiences of discrimination in the healthcare system emerged as themes that were later incorporated into the interview guide. After 19 interviews we concluded that we had reached a saturation of themes. Interviews were transcribed and analyzed using Braun and Clarke’s thematic analysis [28–30]. This inductive method of analysis provided a framework to identify patterns of meaning (themes) within our data, while remaining flexible and organic. We used thematic analysis to produce informed interpretations of our data but unlike in the commonly used grounded theory methodology, we did not develop a theory based on our results. We read the data through our mix of expertise, including lived or living experience, however we acknowledge that themes were filtered through our experiences, knowledge, and aspirations for this work. For a practical, step-by-step [31] approach to thematic analysis we referred to Maguire and Delahunt’s guide which describes thematic analysis as a method, not a methodology, and as such is not tied to a particular epistological or theoretical perspective. After interviews were complete, the authors familiarized themselves with the data, generated initial codes, then themes were coded based on related meaning and merged into various groupings, re-defined, and finalized all with input from MB, MH, SW, and EC. This is as per Braun and Clark’s six-phase method [28].

## Results

Interviews revealed multiple themes, including federal exemptions to prescribing practices, switching to tele-medicine; helpful and/or missing services, the HRM housing crisis, and panic drug buying. We also report findings on our topic of direct questioning, *conceptions of safe supply*. Even though our aim was to explore the views and experiences of people who use drugs and HCPs during COVID-19, especially regarding access to a safe supply of drugs, the interviews expanded to encompass a wide range of issues that both deepened and complicated our understanding of safe supply.

### Conceptions of safe supply

Conceptions of safe supply were elicited in all interviews. Of note, conceptions of safe supply among HCPs were more in-depth compared with people who use drugs who expressed relative unfamiliarity with the concept, which is not unusual in the local community context of the people who use drugs.

A divide exists within the community of addiction medicine practitioners on the subject of safe supply and this was reflected among the HCP we interviewed. However, all but one HCP was supportive of safe supply in our study. The positions of individual practitioners on either side of the divide, ‘for’ or ‘against’ safe supply were nuanced, as illustrated by this statement from a proponent of safe supply:

*“On a broader system perspective*, *I don’t have evidence that tells me what I’m doing is not going to cause harm for the greater population*. *Like am I going to inadvertently shift the drug market here [by prescribing a safe supply of drugs]*?*”–*HCP, addiction medicine physician.

Another HCP expressed hesitation and discomfort with the idea of prescribing a safe supply of drugs without an evidence base to support its use or a dire enough environment to necessitate its use:

*“[Safe supply] spread very rapidly without a lot of rigorous evidence behind its safety… I’m leery about myself being a safe supply provider without having rigorous clinical trials backing the practice*. *I think what’s kind of caught fire with regards to safe supply is because it’s a necessity*, *like in the inner city of Vancouver*, *where it’s the only intervention that will save some lives*. *But I don’t think that’s the truth for the entirety of the rest of the country*.*”* -HCP, addiction medicine physician.

Another HCP argued that much evidence exists out of British Columbia and Ontario supporting safe supply, and to say that we need more evidence in our local context is a “stall tactic”. They likened it to not using a cardiac medication in Nova Scotia if the clinical trial was done in another country. They added, “there is a place for evidence-based medicine and a place for clinical judgement”. Among HCPs who offer safe supply, none reported an adverse event (overdose events or death) among the patients for whom they had prescribed safe supply during COVID-19 (including opioids, stimulants, and/or benzodiazepines).

There was a common consensus among HCPs that neither option (safe supply provision or not) feels “good”, but felt the impact of doing nothing (not offering safe supply) is often worse. Multiple HCPs shared concerns around prescribers providing a safe supply without formal training in addiction medicine and/or a trusting relationship with the patient. A few HCPs felt that an authentic safe supply should consist of a legalized and regulated supply of drugs, and all agreed that layering other health and social services such as increased housing, income, and rehabilitation supports is essential. One HCP described safe supply as a trusting relationship and a human connection that “meets people where they are”, another described a success in safe supply provision as “keeping someone alive one more day, so the next day they can chose the same or something better”. Overall, the majority of HCPs in our study were planning to start or were already prescribing a safe supply of drugs to individuals on a case-by-case basis.

Most HCPs agreed that OAT is a separate discussion and was not considered safe supply by many participants. It was recognized that OAT alone does not help everyone remain safe, such as those who continue to use other drugs while on OAT, and this population in particular needs a safe supply of drugs:

*“That’s what safe supply is really about*. *Recognizing that some people cannot give up the needle and will not be able to and it’s not acceptable to ask them to*, *so what can we do to make that as safe as possible*?*”*–HCP, pharmacist.

Conceptions of safe supply among people who use drugs often turned into discussions around OAT. Some felt that medications like methadone and buprenorphine were a poor solution to addiction, and referred to it as “liquid hand cuffs” as OAT medications are also addictive and often require daily dispensing and/or witnessed ingestion [32]. When asked about safe supply that did not include OAT, many were unfamiliar with this concept, but all expressed, in one way or another, that safe supply would help to keep people alive. Some people felt safe supply was a necessity for people living with chronic pain. One participant described feelings of remorse for leaving their house during lockdowns to access a street supply of drugs, exposing themself and others in their household to COVID-19. In this case they felt safe supply would have been an excellent example of harm reduction. Another person shared their thoughts on safe supply:

*“I think [safe supply] is amazing*. *Because people like me and other women like me who are selling our bodies for drugs wouldn’t have to sell much*. *You know what I mean*?”–Person who use drugs, 38-year-old.

### Barriers and facilitators of safe supply

Some barriers to safe supply from the HCP perspective included fear of potential college repercussions, going “against the grain” of an opioid de-prescribing culture, poor education in professional programs on safe supply, burnout and limited time to spend learning about safe supply, political and moral issues tied to substance use, and fear of causing harm via overprescribing leading to overdose and/or diversion. One HCP felt that safe supply is out of reach as currently “we can’t even get healthcare providers to recognize addiction as a medical problem, let alone provide a safe supply”.

Another barrier to prescribing safe supply relates to the criminalization of drugs in Canada. One participant (HCP) felt the recent regulation and decriminalization of cannabis should be swiftly followed by regulation and decriminalization of opioids as well:

*“Don’t put [safe supply] on the shoulders of doctors and say now you must make this a medical thing*. *We’ve used drugs since the beginning of time and we will always use drugs*, *and what drugs are used will look different but can we just decriminalize this already*, *and try not to medicalize it*? *[Drug use] is real life*, *but it’s also this taboo thing that’s always been with us and we just need to accept it and have some understanding*.*”–*HCP, addiction medicine physician.

One HCP described how previous, less accepted methods of practice, such as extending carries (take home doses of medication), or offering carries in the first place, were occurring more often during COVID-19 as a necessary response to social distancing directives. However, it is worth noting that this arose out of necessity among select physicians, not as a result of the federal exemptions put in place (see sub-theme below, “Federal Exemptions”). One participant proposed bringing together a community of practice specifically for prescribers of safe supply:

*“We have to be housed somewhere because right now there’s a bunch of clinicians practicing addiction medicine and serving people who use drugs and we don’t have a united place where we can all speak*.*”–HCP*, *addiction medicine physician*.

Among people who use drugs, some shared concern about the risk of misuse, such as diversion, as a potential barrier to safe supply. Almost all people who use drugs described stigma and discrimination as an additional barrier to accessing safe supply while only some mentioned the current criminalization of drugs as another barrier. Some felt it unlikely that safe supply could be a legitimate option due to criminalization of substances and mistrust between people who use drugs and HCPs. For example, one participant who lives with anxiety received a recommendation to start on pregabalin by a pharmacist. When the patient brought this up with their family doctor they were met with hostility:

*“You could tell he was not a fan of anybody asking for anything*. *He said stuff like “do you really want a pill for every ill” … he was belittling me … I said could you please just talk to the pharmacist before you go off on me*. *He called the pharmacist*, *and he made sure to say with extra emphasis that I was seeking extra meds*.*”–*Person who uses drugs, 29-year-old.

Another participant described being met with similar antagonism:

*“I was severely depressed*, *and [my doctor] had me on every anti-anxiety/depression medication and after doing the amount of time they suggested for them to work*, *I would keep telling her ‘these aren’t working*, *could we try something else*?*’ And she eventually said ‘listen I know you’re just trying to get drugs from me and it’s not going to happen*, *just cut the bullshit*.*’”–*Person who uses drugs, 21-year-old.

The benefits (facilitators) of safe supply, as described by people who use drugs, included the prospect of saving the lives of people who could otherwise fatally overdose from a tainted supply:

*“I’m sure a lot of my friends would still be here if we had a safe supply*. *I’ve lost so many friends due to this stuff it’s just insane*.*”*–Person who uses drugs, 29-year-old.

### Federal exemptions

In March 2020, the federal government used its powers to exempt prescribers and pharmacists from certain restrictions relating to “controlled substances” under the CDSA, making prescribing and dispensing certain substances more flexible under a certain set of conditions. This allowed physicians to provide prescriptions over the phone and allowed pharmacists to extend and renew prescriptions for controlled substances such as opioids. People who use drugs were also able to have prescriptions delivered to them. Speaking with people who use drugs, however, revealed that many were unaware of these exemptions. One HCP, an addiction medicine physician, explained how the exemptions themselves did not facilitate safe supply as per CAPUD’s definition:

*“[The exemptions] simplify my life a billion times from an administrative and logistical standpoint*, *but how we can use a triplicate [prescription pad] versus a telephone to do a prescription now… that is not the barrier to safe supply at all*.*”–*HCP, addiction medicine physician.

She also described how physicians were prescribing more take home doses of OAT medications, filling larger prescriptions, having fewer scheduled appointments, and requesting fewer urinary drug screens during COVID-19 independent of these exemptions. She described that, for the most part, the changes that arose in healthcare out of necessity during COVID-19 reinforced what she and other addiction medicine physicians were already advocating for and doing in their practices. One HCP shared hope that the pandemic may accelerate positive changes for people who use drugs and improve access to safe supply. But it was clear that the CDSA exemptions introduced during COVID-19 were, by themselves, insufficient to securing safe supply for many people who use drugs.

### The switch to tele-medicine

While tele-medicine may be more accessible for a certain population, not everyone has access to internet or other required technology. Some participants (not restricted to those without technology) commented that their doctors were inaccessible during the switch to tele-medicine. One participant described a feeling that was shared by others in this study:

*“It’s almost impossible to get a hold of a doctor*, *or get an intake*, *or get in to see anybody*. *So*, *you’re dealing with being over the phone*, *all that fun stuff*. *I find it extremely frustrating*, *even now with my surgery*, *to talk to my own doctor has been a nightmare*. *I find basically we’ve just been ignored*.*”*–Person who uses drugs, 35-year-old.

Later, the same participant shared that they were not able to get in contact with their doctor when they had a leg abscess due to an infected injection site:

*“I went without antibiotics for over a week until my leg was to the point of seeping*, *abscessing… I could have died of a blood infection*.*”–*Person who uses drugs, 35-year-old.

She was finally seen by the Mobile Outreach Street Health (MOSH) service a week later and was treated with an aggressive antibiotic, highlighting how injection-related infections [33] are just one of the harms that come with not having access to healthcare or harm reduction services [33].

Similarly, HCPs mostly felt that care was not ideal via the telephone during the early stages of the pandemic. One HCP explained:

*“The greatest challenge has been the disconnect* … *Because that’s the crux of harm reduction*, *of the work*, *is that connection*. *Meeting people wherever they’re at in their lives*. *That contact*. *That engagement*. *That dialogue*. *That respect*. *That care*, *compassion*, *respect and dignity that is so integral*. *So that has been very challenging for people*. *And for us*, *doing our work*. *We miss the people*. *We miss that*. *So [switching to telemedicine] has been a challenge*.*”*–HCP, program lead.

### Helpful and/or missing services

The HRM has multiple OAT clinics around the city, only one OPS, one needle and syringe program, and one withdrawal management center. As these services were not deemed essential, many were closed, albeit some only for a short (1–4 weeks) period during the pandemic. This impacted street-involved clients, who collectively are at higher risk of contracting not just COVID-19 but other drug use-related infections or illnesses [34].

*“We did feel really worried about that (spreading COVID-19)*. *So*, *we tried to minimize a lot our contacts with people*, *but we still found it was a really delicate balance because we thought in some ways the risk was worth it to be out and about because COVID isn’t the only cause of death*.*”*–HCP, mobile health service.

Services reported as most helpful during COVID-19 included MOSH, Barry House, CAPUD, Direction 180, Open Door, local foodbanks, Stepping Stone as well as Mainline Needle Exchange (see Appendix 1 for description of these services). The latter organization is a needle and syringe program with a user-directed and grounded approach. During interviews, participants described positive experiences with Mainline Needle Exchange more frequently than any other service, with examples such as: learning how to inject safely, being connected with helpful resources, and receiving safe injection supplies, among other forms of support such as daily dispensing of cigarettes:

*“Mainline for example*, *they are handing out harm reduction things*. *And during the pandemic they started giving a lot of pharmacies essentially little gift bags of drug use equipment that you would need…which has been really helpful*.*”–*Person who uses drugs, 26-year-old.

Their closure, although brief, made using drugs more dangerous for people who use drugs in the community and in the hospital as well. Mainline’s services include dropping off kits with injection supplies to hospital in-patients at the request of medical staff. In Nova Scotia, there is no formal inpatient addiction support service. While an informal hospital Addiction Medicine Consultation Service has formed to try to address this gap in care [35,36], participants still reported dismissive and harsh treatment in hospital and many participants described avoiding mainstream healthcare due to drug related discrimination.

One interviewee, a HCP who is part of the Addiction Medicine Consultation Service, shared that they received an increase in consults during COVID-19. They commented:

*“My dream is that eventually we’ll have a fully resourced*, *professional consultation service at the [local hospital] and be able to provide that [care] around the clock*.*”*–HCP, resident physician.

One particularly important service that shut down indefinitely and unexpectedly during the pandemic (June 2020) was the only OPS in Atlantic Canada (originally called “HaliFIX”). It did not re-open (under the name of “ReFIX”) until August 2020, leaving many people who use drugs without a safe space to use for months [37]. Some participants did not know about the OPS, its closure, or its re-opening in a new location. An interviewee involved in the OPS’s operations observed that very few females used it after it re-opened as ReFIX. One participant expressed concern for women’s safety with regards to the new location as it borders on a men’s shelter:

*“A lot of these women are escaping abusive relationships and drug addicts and for them to have to go down there and face that*, *I didn’t think it was an ideal spot [the new location]… This is coming from a woman that was involved in prostitution and abusive relationships*.*”*–Person who uses drugs, 35-year-old.

### HRM housing crisis

Participants cited housing instability as a major area of concern. During the initial stages of the pandemic, sweeping efforts were made to house those who were homeless and many Nova Scotians were housed in hotels. One HCP criticized this move, suggesting that there are more efficient ways to spend public funds than housing people in “expensive hotels”. Additionally, some HCPs shared concerns that if people who use drugs were using alone in a hotel room it could increase the risk of overdose deaths (38,39).

One participant in this study, who was living in a hotel at the time of the interview said they had been moved around many times, and this was made more difficult with their physical disabilities and being far away from their OAT clinic. They were worried about what would happen once the government stopped paying for their hotel room as some shelters do not accept people who are actively using drugs and many shelters were at capacity to begin with. A different participant shared:

*“Homelessness is real here in Halifax and especially for women*. *There’s not a lot of opportunities for us*. *Especially when we’re drug addicted*. *As soon as they find out you have an addiction problem*, *you’re a monster*.*”–*Person who uses drugs, 38-year-old.

With a relatively recent boom in real estate prices in HRM, trying to find affordable housing has become increasingly difficult [38]. This proves very challenging for people on a fixed income, like the majority of people who use drugs in this study. One participant, a HCP, explained the unhygienic reality of low-income housing to one of her patients:

*“When you can’t pay more than $600 per month in rent*, *you’re going to have thousands of roommates in the form of rodents and insects and bedbugs and all that*. *And that’s just the way it is*. *You have to learn to live with those rodents and insects”*.*–*HCP, nurse.

### Panic drug buying

Participants reported an increase in the price of opioids during the pandemic. We hypothesized that this was because the supply was restricted by border closures, limiting access to a secure supply of drug ingredients (precursors) and increasing chances of being caught either carrying or trafficking drugs. However, as one HCP explained, a further factor we had not considered was that the opioids purchased on the streets in pre-pandemic Halifax were diverted prescription drugs:

*“The street supply of drugs*, *or prescription supply of drugs really dried up*. *Halifax has historically been a prescription opioid town*. *If somebody has opioid use disorder in NS*, *they are historically addicted to prescription drugs*. *What’s happening is because the doctors weren’t able to provide care or prescribers were providing really acute emergency care*, *the street supply of prescription drugs really dried up*. *That was our way of having a safe supply*.*”–*HCP, Pharmacist.

For some, diverted prescription opioid prices increased so much that people who use drugs ended up using fentanyl instead, as it had become cheaper than the remaining street supply of prescription drugs. According to interviews, the cost of stimulants also increased. On top of this, with the release of the CERB cheques, many people who use drugs noted a concurrent increase in panic buying (purchasing larger amounts of drugs than they normally would), which further deteriorated the supply of drugs, changing our local market:

*“[People are] straight up pulling out $2000 and buying everything that they can because they can at the time*. *Not that I’ve done that specifically but there’s been a lot of panic buying for people and their supply*. *People that usually have sold their prescriptions no longer do because they’re so afraid of running out*. *So*, *it’s just leading to this panic*.*”*–Person who uses drugs, 21-year-old.

With higher prices and an elusive supply, some people who use drugs reported difficulty accessing drugs. One HCP pointed out that drug dealers were facing difficulty being discreet about selling drugs as the streets were mostly free of pedestrians or traffic. Almost all people who use drugs expressed a fear of fentanyl contamination within their supply of drugs, a concern exacerbated by the fact that there are no drug testing facilities or safe supply clinics in Nova Scotia:

*“People are being forced into a situation so desperate that they’re going to that purple fentanyl of unknown purity and source*. *It could even not be fentanyl it could be carfentanil and that really sucks about this pandemic*, *everybody’s got money and everybody’s looking*, *everybody’s getting money around the same time and it’s just a free for all*. *I have both witnessed and heard of overdoses that have occurred because of it*.*”*–Person who uses drugs, 21-year-old.

These pandemic-related changes led to people who use drugs using more dangerously—in isolation, with an increasingly precarious supply and a lack of social support services and safe using supplies [39]. Inevitably, this put people at increased risk of both fatal and nonfatal overdoses. Nova Scotia has seen a consistently high number of opioid overdose deaths during COVID-19 [40].

*“I do know that heroin is a lot more dangerous*, *there’s the fentanyl involved with it*, *and I experienced*, *for the second time*, *I overdosed*. *And I had a friend who overdosed*. *So that’s something that’s changed during the pandemic because I wasn’t used to people overdosing… you don’t know what’s in it [street drugs]*, *it’s so easy for people to overdose*.*”*–Person who uses drugs, 26-year-old.

## Discussion

People who use drugs face several social and health challenges which only worsened during the COVID-19 pandemic. We found that access to services, housing support, and interventions to use safely were lacking during COVID-19 in HRM. As many challenges faced by people who use drugs exist outside of COVID-19, we argue that it would be unethical to retract the community interventions and changes in practice that were made to support people who use drugs during the pandemic, such as prescribing a safe supply of drugs.

This study had two main limitations. First, we did not collect or analyze any quantitative demographic data on gender, ethnicity, or sexual orientation. Second, our findings are context specific and drawn from a small sample of interviews, such that they may therefore be difficult to extrapolate from, but this is the first qualitative study related to safe supply in our local context. The main strength of our study is that it centers the voices of people who use drugs, whose expertise is consistently absent from research, service design and delivery, and policy-making—locally, provincially, and nationally [11]. This historical gap in research scope and design negatively impacts the quality of harm reduction services, including safe supply, amidst the ongoing syndemic of COVID-19, HIV, hepatitis C, homelessness, and a toxic drug supply.

Our research participants, particularly those who identified as people who use drugs, were united in the view that communities know best how to solve their own problems, and that political will and financial support are the most significant obstacles to designing and implementing community-led responses to the most pressing issues affecting people who use drugs.

This is especially true with respect to the design and implementation of safe supply. In places like Vancouver or Toronto, safe supply was being prescribed by physicians prior to the pandemic and by prescribers in the HRM, but to a much lesser extent [17,41]. Yet not all safe supply initiatives have been providing robust formal supports, in terms of intentional participatory design processes, facilitative regulatory mechanisms or funding. In 2021, a safe supply project in Victoria, BC received four million dollars to operate over the next three years. This project, SAFER, which has two locations, one in Vancouver and the other in Victoria facilitates access to more progressive prescribed pharmaceuticals such as fentanyl, sub-lingual fentanyl, and fentanyl patches as an alternative to the contaminated unregulated drug supply [42,43]. In contrast, smaller or more rural areas such as the Atlantic Region (NS, New Brunswick, Prince Edward Island, Newfoundland) only offer safe supply via a prescriber and their patient on a case-by-case basis, without formal support from the College of Physicians and Surgeons. Meanwhile, HRM’s street supply of drugs is seeing more and more fentanyl and fentanyl analogues mixed with benzodiazepines such as flurazepam and etizolam. When used in combination, these drugs present a high risk of fatality if not used properly as they all decrease respiratory effort [44–46].

Our findings nevertheless underscore several areas of ambiguity surrounding the meaning of safe supply, which is consistent with previous research [11,47,48].

The first concerns the scope of substances understood to constitute safe supply. While CAPUD’s definition of safe supply encompasses a broad range of drugs, it expressly excludes those that aim to reduce drug use and/or treat drug related dependence, such as OAT. Yet, one of the prescribers and two participants in our study disagreed with this conceptualization and regard OAT as an essential part of safe supply.

Secondly, the mechanism or mechanisms by which safe supply is provided is not settled. The consensus among HCP in our study was that safe supply should be provided on a case-by-case basis by skilled addiction medicine physicians. In our study, two of three healthcare providers with prescribing privileges were prescribing a version of safe supply in their practices prior to the pandemic. Most HCPs we interviewed had difficulty envisioning, or outright opposed, the provision of safe supply outside of a prescriber-patient relationship. People who use drugs, in contrast, generally expressed enthusiasm for the idea of safe supply but, in many cases, unfamiliarity with the concept and its scope. This disconnects between the level of understanding and related consolidation of opinion among HCPs on the one hand, and people who use drugs on the other, indicates first the exclusive or near-exclusive space occupied by medical professionals in shaping safe supply in line with their comfort zones and preferences, and second, the absence of people who use drugs in informing policies and processes relating to service design and delivery, including those oriented to safe supply. This suggests a missed opportunity to draw upon a wide range of experiences and opinions, inclusive of those whose fundamental interests are most affected, in determining whether or how to expand the meaning of safe supply to include access to a broad range of drugs, possibly with minimal prescriber supervision, under conditions that diverse people who use drugs experience as safe.

Therein lies the third, and final, area of ambiguity: the conditions under which safe supply is accessed. Similar to a syndemic, our participants’ experiences and insights show how safe supply must be understood in intersectional terms. For example, where safe supply is accessed matters depending on the people involved: for women who use drugs, the relocation of the OPS to a site neighboring a men’s shelter rendered it unsafe given the abuse some women in our sample had previously endured from some of the men living there. For people who use drugs with HCP relationships that are characterized by stigma, rooting safe supply in the same relationship is, too, going to make the experience of accessing safe supply feel less safe. Also, for people who use drugs that are experiencing homelessness, accessing healthcare in general is a challenge requiring creative models of delivering a safe supply of unadulterated drugs that is accessible to those who may benefit most.

The question moving forward is how will these areas of ambiguity come to be resolved. In our view, meaningful integration of people who use drugs is essential to shaping the scope, model, and conditions of safe supply in Nova Scotia.

With the perspectives of people who use drugs at the forefront of our study, we learned of the many threats to safety and wellbeing that were worsened by the pandemic. The rapid switch to telemedicine was met with mixed reviews in our study with an overall consensus that it was challenging to both HCPs and people who use drugs. Much of the criticism stemmed from the growing pains of implementing a new system on short notice as well as the reported unavailability of virtual appointments during early stages of the pandemic. This intervention has the potential to greatly improve access to healthcare, but if used without discretion, may lend toward subpar care–a concern from some HCPs in our study.

The stigma and discrimination faced by people who use drugs when accessing healthcare, as reported in our study as well as in the literature [7,8], is independent of COVID-19 and speaks to the urgent need for change in our current model. Ideally, the informal addiction medicine consultation service in HRM will become formalized to address this gap in the system (31).

A major concern among the people who use drugs in our study was homelessness or insecure housing, despite many individuals being rapidly but temporarily housed in hotels during the pandemic. People living on the streets were underrepresented in our study, as this population is much more difficult to connect with, especially with the social distancing requirements imposed at the time our study took place. Nonetheless, we highlight the need for further housing supports as we know many people who use drugs are forced to live on the streets, and this is exacerbated by “zero tolerance” policies for substance use in most local shelters. Homelessness can preclude individuals from receiving a safe supply of drugs or carries (take home doses) with OAT, as they have no safe space to store their medications. On top of this, people who are injecting drugs in the streets are in less safe environments and at higher risk of acquiring injecting-related infections such as infective endocarditis, sepsis, and skin abscesses [49].

Eventually, a group of HCPs, including doctors, nurses, pharmacists, and community workers in HRM provided 77 precariously housed individuals required to isolate in a hotel due to community related COVID-19 exposure with a wide ranging medicalized safe supply of drugs, such as pharmaceutical alternatives to opioids, benzodiazepines, and stimulants as well as alcohol and cigarettes. This intervention lasted 25 days and the results showed low rates of adverse events (overdose, intoxication, diversion, sharing, or selling of safe supply medications or alcohol) and high rates of successful completion of the mandatory 14-day isolation, suggesting this intervention was safe and effective [50,51]. This intervention shows promise for providing safe spaces for individuals who are experiencing homelessness as well as using drugs.

The federal drug exemptions did not obviously improve care for people who use drugs in our study. HCPs commented that it did not assist people who use drugs in HRM during COVID, other than by simplifying administrative burden on HCPs that may indirectly free up prescriber time to see more patients. Within our cohort, however, some HCPs were making exceptions and prescribing substances (safe supply) out of “sheer necessity” within a trusting prescriber-patient relationship, with or without back up from the college.

People who use drugs who were not able to access a safe supply of drugs, which was the vast majority in our study, reported major changes in the drug supply. This is in line with the findings of Ali and colleagues (2021) who describe major disruption in supply during COVID-19 and an increased risk of overdose as a result [52]. In addition, we noted increased fentanyl use because of a relatively cheaper cost and panic drug buying as an effect of CERB payments. The changes in drug supply and purchasing, as well as the use of more potent substances made using substances particularly dangerous during COVID-19.

Community service availability may have lacked emergency preparedness. Many services were forced to shut down or closed briefly during certain stages of COVID-19 lockdowns and the people who use drugs in our study suffered the consequences of not being able to access safe injection supplies, community supports, housing, or access to food via food banks. This was recently corroborated in a national study [53]. It is worth considering whether some of these services should be deemed as “essential” moving forward.

## Conclusion

In conclusion, people who use drugs in HRM are in the same crisis as the rest of Canada, including a wide variety of health and social issues contributing to the syndemic of high rates of overdose, COVID-19, homelessness, HIV, and HCV. As the COVID-19 pandemic remains, the drug supply continues to become more potent with deadly toxins. People who use drugs continue to lack access to the basic supports required to meet their most fundamental human needs, a problem that pre-existed the COVID-19 pandemic yet has been exacerbated by it. In 2022, Nova Scotia reported 62 overdose deaths, implying that our interventions have not been sufficient [40]. The voices highlighted in this research urge Nova Scotia to do more to combat the syndemic experienced by people who use drugs. Additionally, an overwhelming number of people who use drugs in our study described feelings of stigma and discrimination when accessing healthcare services and these experiences were only exacerbated during COVID-19 as people who use drugs tried to secure a safer supply of drugs [8]. This stigma and discrimination can lead to tragic outcomes via the avoidance of healthcare altogether. We need to ask ourselves, since “doing nothing feels worse”, how to best support access to a safe supply of drugs as a first step toward demonstrating equal respect for the lives, liberty, and security of people who use drugs.

## Supporting information

S1 Appendix (DOCX)

S1 File (DOCX)

S2 File (DOCX)
