# Supplementary material for: ’More of the same, but worse than before’: A qualitative study of the challenges encountered by people who use drugs in Nova Scotia, Canada during COVID-19
Source: PLoS One. 2023 Apr 5;18(4):e0283979. doi: 10.1371/journal.pone.0283979 (PMC10075390; doi:10.1371/journal.pone.0283979)
Supplement: S1 Appendix — (DOCX) [file pone.0283979.s001.docx]

**Appendix 1**

Mobile Outreach Street Health (MOSH): MOSH provides accessible primary health care services to people who are homeless, insecurely housed, street involved and underserved in our community. http://moshhalifax.ca/

Barry House: Barry House is a 17-bed emergency shelter serving individuals who identify as female or are gender diverse. <https://www.shelternovascotia.com/shelter-services>.

Canadian Association of People Who Use Drugs (CAPUD): The Canadian Association of People who Use Drugs (CAPUD) is a national organization that is comprised entirely of people who use(d) drugs, including our board and staff. One of our main purposes is to empower people who currently use drugs deemed illegal to survive and thrive, with their human rights respected and their voices heard. https://www.capud.ca/

Direction 180: Direction 180 is a community-based, opioid treatment program. <https://direction180.ca/>.

Open Door: An opioid replacement and family practice clinic serving Dartmouth, NS since 2016. [www.theopendoorclinic.ca/tod/index.html](http://www.theopendoorclinic.ca/tod/index.html)

Stepping Stone: Stepping Stone is the only non-profit organization in the Maritimes that supports women, men, and transgender persons currently and formerly involved in the sex trade by contributing to their health, safety and well-being. <https://steppingstonens.ca/about/>

Mainline Needle Exchange: Mainline, a program of the Mi’kmaw Native Friendship Center, is a health promotion organization dedicated to supporting PWUD through harm reduction programs. <https://mainlineneedleexchange.ca/>
