# Supplementary material for: ’More of the same, but worse than before’: A qualitative study of the challenges encountered by people who use drugs in Nova Scotia, Canada during COVID-19
Source: PLoS One. 2023 Apr 5;18(4):e0283979. doi: 10.1371/journal.pone.0283979 (PMC10075390; doi:10.1371/journal.pone.0283979)
Supplement: S1 File — (DOCX) [file pone.0283979.s002.docx]

**Transcript Excerpts**

*“On a broader system perspective, I don’t have evidence that tells me what I’m doing is not going to cause harm for the greater population. Like am I going to inadvertently shift the drug market here [by prescribing a safe supply of drugs]?” –* HCP, addiction medicine physician.

EC: Okay, that wasn’t really what I was expecting but that makes a lot of sense. We’ll just move on to the stage 3 questions. So, what you just said, these logistical things aren’t the barrier to safe supply, and you mentioned what I interpreted as some barriers, like how people worry there’s too much prescribing of opioids, since we worked really hard to get it under control, we just need access points with trained prescribers. Also, a bit of fear about college repercussions, would those fall under what you would call the main barriers to safe supply? Are there some I've missed?

004: Yeah, those are the main barriers to safe supply. And the other thing is that there is a huge divide in the addiction treatment community about this right? The physicians who are doing this, to some part myself included, although I’ve definitely experienced less so because I’m doing it in less skilled environments and keep doing it […]. The pharmacist I work with, and they're like “thank goodness you’re doing this, I was really worried about this patient”. There’s a lot of […] out there who know what I do but not a ton of […] who know that I do safe supply for some of my patients. I haven’t had that much backlash, although I’ve had some. When you’re doing things that people worry about college implications, it helps when you have a community of people who are behind you. If you feel you have an evidence base to support what you’re doing - we do that in medicine all the time. So long as we feel we can defend ourselves from an ethical and evidence based perspective we can feel comfortable in what we’re doing even though we’re pushing the envelope on something. I think with safe supply there’s a huge divide in the community of practice of addiction medicine. I think a lot of people, myself maybe included, I know when I have a patient in front of me what the right thing to do is. From a broader system perspective, I don’t have evidence that tells me what I'm doing is not going to cause harm for the greater population. Like could it shift the drug market, am I going to inadvertently shift the drug market here? We don’t have evidence about that. We have lots of assumptions about things, but I think that’s a lot of the fear with physicians and NPs. If we’re going against the grain, and the grain is really vocal, cause sometimes some of the physicians in […]. The conversations get really ugly. If we’re going to go against the grain and get a lot of backlash from people then we want to know, when we’re thinking about the downstream effects, the broader public health and impacts… I think that’s where there’s a lot of fear or discomfort or concern. I think there’s lots of people, if we had a really robust connected community practice across the country that said “okay, let's all do this and measure outcomes”. If we’re embarking on this, my assumption is that, if we’re collecting data on all things and thinking okay I think people would feel better about it. The reality is that all of us are doing this on top of really juggling burnout most days and juggling all the other inefficiencies of the healthcare system that allocations disproportionately suffer from, inequities in all kinds of things, it’s always that for me and the people I work with, it’s always all these projects that need to change in your 25th hour and when you’re juggling burnout and the pandemic and all of these other things and when you see these discussions happening on a broader level it can be really overwhelming. The other thing to say is that a lot of this really, safe supply is important. But decriminalization is super, super, super important. Those are conversations, and it's the same with, where cannabis was approved by health Canada so quickly, when really the answer for a lot of them was to decriminalize cannabis. Don’t put it on the shoulders of doctors and say now you must make this a medical thing. It’s the same with drug use. We’ve used drugs since the beginning of time and we will always use drugs, and what drugs are used will look different but can we just decriminalize this already? And try not to medicalize it, it's real life. I think that’s the other piece too, that some may be struggling with the intersect that this is a broader social issue, not just a medical issue. Yes it’s a medical issue that I can have an impact by giving this person safe supply, but this is a very small piece in terms of what needs to change in our perspectives about PWUD, drug culture, you know, cause like it’s this taboo thing but really it’s always been with us and we just need to accept it and have some understanding.

*“[Safe supply] spread very rapidly without a lot of rigorous evidence behind its safety… I’m leery about myself being a safe supply provider without having rigorous clinical trials backing the practice. I think what’s kind of caught fire with regards to safe supply is because it’s a necessity, like in the inner city of Vancouver, where it’s the only intervention that will save some lives. But I don’t think that’s the truth for the entirety of the rest of the country.”* -HCP, addiction medicine physician.

EC: My next question is, what are your thoughts on providing a safe supply of drugs. Opioids specifically.

017: It’s a contentious topic and it’s a practice that seems to have spread very rapidly without a lot of rigorous evidence behind its safety. That’s my general opinion.

EC: Okay, that seems to be the common thread. It’s super complicated. And the other thing I’ve been hearing is that it should only be provided by a select few that are really experienced, is what I’ve understood.

017: I’d go a step further, I think restricting it to people who have expertise in addiction is certainly one thing but I’m leery about myself being a safe supply provider without having rigorous clinical trials backing the practice. I think what’s kind of caught fire with regards to safe supply is because it’s a necessity, like in the inner city of Vancouver, where it’s the only intervention that will save some lives. But I don’t think that’s the truth for the entirety of the rest of the country.

EC: Because the drug supply is so much less tainted in HRM for example compared to Vancouver?

017: What I see too is that the future of substance use in the HRM and NS is likely to evolve. There may be a day where we face just as many problems as exist in the downtown east side and when that day comes I’d be ready with every intervention we have, including this if we have rigorous research behind it.

EC: Yeah, what is your biggest… what would you be most concerned about in providing a safe supply?

017: Introducing additional drugs to the community at large. I’m worried about diversion. I’m also worried about what safe supply might be doing to the individual from a medical standpoint. I’ve seen some early indications that injecting pills that weren’t meant for injection can lead to additional health consequences like endocarditis.

*“That’s what safe supply is really about. Recognizing that some people cannot give up the needle and will not be able to and it’s not acceptable to ask them to, so what can we do to make that as safe as possible?”* – HCP, pharmacist.

EC: Right, interesting. Another thing I learned through CAPUD is how safe supply in general isn’t what I thought it was. For example, methadone isn't a safe supply. And when you center the patient experience of that “warm hug” or high in general, that’s the safe supply.

005: Yeah, safe supply isn’t getting everybody on methadone and suboxone and moving forward. SS is providing pharmaceutical grade opioids, heroin included. They did it in Portugal. That's why I encourage you to read chasing the scream. Basically, in Portugal in 2001 they have an unbelievable rate of heroin addiction. 10% of the population was hooked on heroin, of the entire adult population. That’s wild. So that means if you’re not addicted to heroin, someone in your family or friends is. Chances are they're not working, and chances are you in some capacity are having to take care of them. So, the economy ground to a halt and the government looked at this and though this isn’t working what we’re doing, just arresting people and putting them in jail just hasn’t worked. We’re just increasing the number of people who are in jail and hooked on heroin. The definition of insanity is doing the same thing over and over again and expecting a different result - Albert Einstein. It’s time to do something different. So, what they did was say frig this, let’s decriminalize. They decriminalized personal supply, a reasonable supply for 10 days’ worth. You couldn't get arrested for that. They took all the money that they were spending on arresting people and incarcerating people and put it into safe injection sites, safe consumption sites, and safe supply. They set up heroin clinics everywhere. All these people really can’t stay on methadone or OAT in any way, they're just not going to ever. That exists everywhere. That exists in smoking cessation, you can get people onto a vape but they’ll be on a vape forever. My [relative] is on nicotine gum and has been chewing it for 15 years. She’s never going to not chew nicotine gum, it’s just not going to happen. It’s also just not a reasonable goal. So, they said well let's just get people safe, who cares. They also put money into employment programs where they said alright the people who are in these heroin and addictions programs, we’re going to pay employers to hire them for one year. We’ll pay their salary but they work for you for a year. If after that you like them you can hire them on. And some crazy stat, I forget what it was, 75% of people were hired on afterwards. Even though they were “a junkie” I hate that word, still using it, but it was a safe supply, they don’t want to be high all the time. They don’t want to be living that life of living on the street, they want to have a life they just have this learned behavior and trauma in their lives that doesn’t allow them to. Safe supply is really looking at the entire picture and thinking okay we could get people on methadone in the program, and that's a form of safe supply, but we’ve got people that will never no inject and not use. They just can’t. So, let’s give them something that will not kill them. What we saw in Portugal is a quick uptick in access to drugs, you’re getting heroin for free, sign me up, and people’s usage went up quite significantly. Then it petered out and people were like okay that was fun, I'd like to get back to life now and it went back down to below baseline. There is evidence for that, for safe supply. Safe supply can even mean a family doc or NP who has a patient they’ve tried on methadone 7-8 times and it just won’t work, and they think what can we do here, let’s prescribe you injectable hydromorphone and see what happens. Chances are, what you see in the literature and in Portugal, they’re going to be awesome and do just fine and be way better than if we didn’t. That’s what safe supply is really about. Recognizing that some people cannot give up the needle and will not be able to and it’s not acceptable to ask them to, what can we do to make that as safe as possible?

EC: What do you think are the main barriers to having a safe supply here? It seems to me it barely exists, and the one of […] providers who are really trying to provide a true safe supply are kind of acting on their own.

005: They’re doing it … because they know it’s the right thing to do. They’re not doing it with any sanction from […] or anything like that. What sucks about substance use disorder is for some reason, similar with abortion debate, which is a ridiculous debate. We’ve taken something that is a health issue and made it a political issue. A moral issue. And that comes from this war on drugs that started in the 50s in the states. They assigned morals to this. You're good if you don’t do drugs and you’re bad if you do. That is well entrenched in our society. When we have millennia of substance use as a human race that was just part of our culture. Pretty much every culture across the globe with all some sort of historical use of some substance for the purpose of pleasure alone. That’s just a known fact. So we’ve taken a health issue and made it into a moral issue. Therefore, if it’s a moral issue, it’s also a political issue in North America. So safe supply we can say it’s an issue of education, which it is, we need to explain to people what it is and why we provide somebody with drugs to use, and how it’s not the same as encouraging drug use and why it’s not going to be encourage some 12 year old kid to pick up a needle where they wouldn't otherwise. That’s not going to happen. It is a matter of education that you can educate somebody until they’re blue in the face. Until you get somebody like Justin Trudeau or somebody willing to provide decriminalization and safe supply and know they probably won’t get elected next term because of it, it won’t happen. That’s because it's become a moral and political issue. Unfortunately, politicians are here for Canadians is what we're told, and have to believe that to get up in the morning, but at the end of the day they’re also here to get re-elected. Until we change the psyche of Canadians to one of “cool this is a learning behavior, a learning disorder, trauma-informed care piece, a health issue and here’s the best available evidence to treat it” which it is, and have them accept that, it’s going to be an uphill battle. I was at a conference a couple years ago and the […]was there and I asked “can you explain why we don't have safe injection sites in Halifax?” and he said well we need to really study it and research it. I raised my hand and said I can find you probably 100 papers based out of Portugal and Vancouver that show that this works. But “we don’t have it in the NS context”. Well if that’s your argument, we also don’t have a study for Ramipril post-MI in NS because that was done in the states by the makers with 100,000 people. So, if we can’t extrapolate from that that we can use it in Nova Scotians, then we need to study everything that we do in NS. That’s what we’re up against. We are being told that we need to study it and study it and that’s really a stall tactic so they don't have to do anything about it.

*“I think [safe supply] is amazing. Because people like me and other women like me who are selling our bodies for drugs wouldn’t have to sell much. You know what I mean*?” –PWUD, 38-year-old.

EC: What are your thoughts on doctors offering a safe supply of opioids.

014: I think that’s amazing. Because people like me and other women like me are selling their bodies for drugs wouldn’t have to sell much. You know what I mean?

EC: Yeah, you’re putting yourself at risk and –

014: big time. Double at risk not only with the drugs but with the John’s [men] too. At the end of the day we don’t know what they’re going to do. We just hope to god they’re going to pay us. Sometimes they do, sometimes they don’t.

EC: Wow. That’s so unfair. So, you would say a safe supply would be a good option especially for people–

014: Yeah as long as it’s monitored. It could go wrong in so many different ways. But at least we know it isn’t laced with fentanyl. There are so many other risks that we’re taking with fentanyl on the street. It’s crazy. Now we’re getting addicted to the fentanyl not the opiates.

EC: Yeah, so in terms of when you said unless it’s monitored. What are your thoughts on urinary drug screens?

014: Oh, for sure. Because then you know exactly what they’re using and doing. But there’s ways around that too you know. Lots of ways. If there’s a will there’s a way and an addict will find it. It’s trial and error, like anything else. I really do think that we do need more support in opiate crisis here in Halifax and in NS period. So many of us are falling on deaf ears, we’ve been asking for help and asking for this/that and there’s just no resources out there for us. And the ones that are there for us are being utilized to the extreme, to where they’re dwindling away to nothing because they’re using so much and so often because it’s the only ones we have.

EC: You’re so right. This is kind of a weird question but I’ve asked it to everyone. If you could give advice to people in power, people who make decisions like politicians or police, what would you say to them?

014: The whole system needs to change. The whole system is broken. I think it needs to be re-written and they need to start listening to the addicts and listening to the people who are in recovery and are struggling with it constantly. I just think the system was made by a person who never dealt with addiction before. They’re just by what they see and read in a textbook, not by what they’re dealing with in real life. Because real life issues are a lot different from a textbook issue.

*“Don’t put [safe supply] on the shoulders of doctors and say now you must make this a medical thing. We’ve used drugs since the beginning of time and we will always use drugs, and what drugs are used will look different but can we just decriminalize this already, and try not to medicalize it? [Drug use] is real life, but it’s also this taboo thing that’s always been with us and we just need to accept it and have some understanding.”* – HCP, addiction medicine physician.

EC: Okay, that wasn’t really what I was expecting but that makes a lot of sense. We’ll just move on to the stage 3 questions. So, what you just said, these logistical things aren’t the barrier to safe supply, and you mentioned what I interpreted as some barriers, like how people worry there’s too much prescribing of opioids, since we worked really hard to get it under control, we just need access points with trained prescribers. Also, a bit of fear about college repercussions, would those fall under what you would call the main barriers to safe supply? Are there some I've missed?

004: Yeah, those are the main barriers to safe supply. And the other thing is that there is a huge divide in the addiction treatment community about this right? The physicians who are doing this, to some part myself included, although I’ve definitely experienced less so because I’m doing it in less skilled environments and keep doing it […]. The pharmacist I work with, and they're like “thank goodness you’re doing this, I was really worried about this patient”. There’s a lot of […] out there who know what I do but not a ton of […] who know that I do safe supply for some of my patients. I haven’t had that much backlash, although I’ve had some. When you’re doing things that people worry about college implications, it helps when you have a community of people who are behind you. If you feel you have an evidence base to support what you’re doing - we do that in medicine all the time. So long as we feel we can defend ourselves from an ethical and evidence based perspective we can feel comfortable in what we’re doing even though we’re pushing the envelope on something. I think with safe supply there’s a huge divide in the community of practice of addiction medicine. I think a lot of people, myself maybe included, I know when I have a patient in front of me what the right thing to do is. From a broader system perspective, I don’t have evidence that tells me what I'm doing is not going to cause harm for the greater population. Like could it shift the drug market, am I going to inadvertently shift the drug market here? We don’t have evidence about that. We have lots of assumptions about things, but I think that’s a lot of the fear with physicians and NPs. If we’re going against the grain, and the grain is really vocal, cause sometimes some of the physicians in […]. The conversations get really ugly. If we’re going to go against the grain and get a lot of backlash from people then we want to know, when we’re thinking about the downstream effects, the broader public health and impacts… I think that’s where there’s a lot of fear or discomfort or concern. I think there’s lots of people, if we had a really robust connected community practice across the country that said “okay, let's all do this and measure outcomes”. If we’re embarking on this, my assumption is that, if we’re collecting data on all things and thinking okay I think people would feel better about it. The reality is that all of us are doing this on top of really juggling burnout most days and juggling all the other inefficiencies of the healthcare system that allocations disproportionately suffer from, inequities in all kinds of things, it’s always that for me and the people I work with, it’s always all these projects that need to change in your 25th hour and when you’re juggling burnout and the pandemic and all of these other things and when you see these discussions happening on a broader level it can be really overwhelming. The other thing to say is that a lot of this really, safe supply is important. But decriminalization is super, super, super important. Those are conversations, and it's the same with, where cannabis was approved by health Canada so quickly, when really the answer for a lot of them was to decriminalize cannabis. Don’t put it on the shoulders of doctors and say now you must make this a medical thing. It’s the same with drug use. We’ve used drugs since the beginning of time and we will always use drugs, and what drugs are used will look different but can we just decriminalize this already? And try not to medicalize it, it's real life. I think that’s the other piece too, that some may be struggling with the intersect that this is a broader social issue, not just a medical issue. Yes it’s a medical issue that I can have an impact by giving this person safe supply, but this is a very small piece in terms of what needs to change in our perspectives about PWUD, drug culture, you know, cause like it’s this taboo thing but really it’s always been with us and we just need to accept it and have some understanding.

EC: Yeah, that’s a really good point. I was going to ask some more questions about what you or a community of doctors would need to feel more supported, would it be the department of health and wellness speaking up? Not feeling like you’re just floating - now that you say this it just opens a whole new can of worms. I’m curious what you think are the most important things you’d like to see done in terms of protecting the health and safety of PWUD in HRM?

004: So many things. I see that as two separate questions. One, what can we do for the personal protection of PWUD in HRM and then the other, what needs to happen to advance discussions about safe supply or broader safe supply discussions and the prescriber’s role in that. So, from a perspective of PWUD, we have some pretty antiquated ideas around here about what that means. I mean it’s changing but I think of the demographic of NS and the geography and all that stuff, and things move slowly here. Sometimes that's okay, and sometimes it’s really detrimental. We need to do a lot to change thoughts and beliefs and attitudes about PWUD and tear down those negative stereotypes in the general population. We need to decriminalize drug use, 1000%. We need to really rethink our incarcerations and why we’re incarcerating people who use drugs or who deal drugs. Just having that carceral approach is not effective and needs to be re-evaluated. The relationship with police here is of course very problematic, particularly for BIPOC in NS. That whole thing needs to be really, really re-examined and defunded. Communities know how to solve their own problems, we need a more civilian-led response to most of these issues that impact PWUD. Things like sobering centers, community org response to mental health crisis. We need police for the things that police are meant to do. We don’t need police involved in things that are better handled by the community, and the community knows what the answers are. We just need to ask PWUD, but they’re not at the table. In the same way we have to look at our relationships with BIPOC people, we can’t tokenize PWUD, we can’t say we have somebody who’s a former drug user and have them as the token drug user at the table. It’s just one person, one perspective, and it’s […] because it’s optical. It’s optical ally ship, it’s not true ally ship. Who’s the author of white supremacy, I don’t remember her name. But I see that in the same way. We have to have true ally ship with PWUD and their experiences, and really the decriminalization piece is huge. If we start there, we’ll understand what we need to do. Instead of, in our places of privilege without lived experience, trying to solve those problems.. Because we don’t know the answers. And making sure that group is diversified. There are people who actively use drugs, who have substance use disorders, who use recreationally, and everything in between. There are PWUD who are indigenous and there are all these stereotypes and layers. We need a diverse voice. So that’s one piece.

*“You could tell he was not a fan of anybody asking for anything. He said stuff like “do you really want a pill for every ill” … he was belittling me … I said could you please just talk to the pharmacist before you go off on me. He called the pharmacist, and he made sure to say with extra emphasis that I was seeking extra meds.” –* PWUD, 29-year-old.

EC: The next question is “is your supply of drugs stable” I guess you just answered that. No, it doesn’t sound like it’s very stable at all.

001: It was before the pandemic. Before the pandemic, if I wanted, I could literally just call somebody. I could catch a bus, be there in 15 minutes, but now you pretty much… if you don’t have someone on dial that you can call, you have to roam around [street name] for hours until you see somebody who might. And it’s depending on if you know them, they may price gouge. It’s certainly very rough at the moment. I feel bad for people who don’t even have Suboxone or methadone because holy shit… yeah.

EC:  So, during the peak of social distancing, would you say most days you’d have to leave your house and go and try to …

001: Yeah. I honestly felt very shitty about that. I wish that, at the time, I could have just gone to my doctor. That’s all it would have took. A note on a pad. And I’m not saying it’s a perfect thing. But if it’s between that and disparity and exposure and risk.. Like going on the bus peak pandemic because that’s the only way you’re going to get something. That is a very dangerous game to play. And coupled with the fact that if you get sick from COVID and you get sick, it’s not going to be good. So yeah, I definitely repeatedly exposed myself to unnecessary risks during the COVID-19 pandemic.

EC: Have you heard about any of the federal exemptions that they made, for example prescribing things over the phone, and allowing pharmacies to drop off medications. Did you benefit from any of these exemptions?

001: The exemptions over the phone and stuff? Yeah. I did get to speak with a doctor over the phone but none of the exemptions to controlled substances or prescribing I never benefited from. I doubt my physician would ever jump on it. Back in February I was trying to get on pregabalin and I wasn’t on anything else at the time and was having massive anxiety issues. I had been going to therapy and I didn’t want to be on benzos at the time. I asked the pharmacist what would you recommend? It seems like pregabalin was somewhat of a good fit. And when I discussed the possibility of it with my doctor he got very volatile and unprofessional very quickly. Maybe not volatile but very.. You could tell he was not a fan of anybody asking for anything. He said stuff like “do you really want a pill for every ill” or when he got on the phone with the pharmacist, because he was belittling me at that time, and I said could you please just talk to the pharmacist before you go off on me. He called the pharmacist, and he made sure to say with extra emphasis that I was seeking extra meds.

EC: I was going to ask, what do you mean you can’t picture your physician jumping on that but I think I understand more now what you’re saying. Earlier you mentioned that when you were in school, you were taking something, a stimulant….

001: Oh Dexedrine?

EC: Maybe that was it. Did that prescription change, was it monthly?

001: That prescription has largely stayed the same. But because I’m not currently in school I’ve reduced the prescription down because I find it gives me anxiety. But he has no problem prescribing anything like that and it’s never been withheld.

*“I was severely depressed, and [my doctor] had me on every anti-anxiety/depression medication and after doing the amount of time they suggested for them to work, I would keep telling her ‘these aren’t working, could we try something else?’ And she eventually said ‘listen I know you’re just trying to get drugs from me and it’s not going to happen, just cut the bullshit.’”.* – PWUD, 21-year-old.

EC: Do you feel stigmatized as someone who uses substances and has it changed at all during the pandemic?

003: Oh incredibly. And no, it hasn’t changed.

EC: Can you think off the top of your head, of a time where you really felt stigmatized, or someone said something, or a HCP or family member or friend said something?

003: Yeah. My doctor, I was severely depressed, and she had me on every anti-anxiety/depression medication and I kept telling her, after doing the amount of time they suggested for them to work, and I would go back to the doctor and I would keep telling her these aren’t working, could we try something else? And she eventually said ‘listen I know you’re just trying to get drugs from me and it’s not going to happen, just cut the bullshit’.

EC: Which is interesting because having to try multiple antidepressants is kind of the norm?

003: Yeah, you’d think. I gave up. She knows I’m an addict and she thought I was just trying to get benzos from her but that wasn't the case I just wanted to die pretty much.

EC: That’s not right. How is your mental health more recently since you’ve been on methadone.

003: A lot better. I can actually get out of bed in the morning. I don’t have to worry about how I’m going to afford to get high today and all that fun stuff.

EC: That’s really nice to hear. So, before you started on methadone, can you walk me through, step by step, what using looked like for you. From acquiring substances to finding a space to use them etc.

003: Yup. Everyday was different and it depended on how much money I had. If I didn't have a lot of money I would just drink, probably a quart of vodka. If I had the money I would get [hydromorphone pills] and do them alone. I started shooting up and didn't know how to, it took a while to learn how to do it properly. That was reckless and stupid of me to be doing that alone, completely alone, I wouldn’t even tell a friend ‘this is what I’m doing’ just in case something bad happened. Probably a 60-80 dollar a day habit towards the end.

*“I’m sure a lot of my friends would still be here if we had a safe supply. I’ve lost so many friends due to this stuff it’s just insane”* –PWUD, 29-year-old.

EC: Can you think of any other services that could have really helped you? Or more generally, when everyone went on lockdown, you were just out of hospital, did you ever feel that you were putting yourself at risk leaving your home to buy drugs? Or did the pandemic not have a big impact on your life considering what you just went through?

003: Yeah it didn’t have that big of an impact at all. I mean it sucked because I was in the hospital for months and months and I finally got out and the next day everything was a big lock down. So yeah it sucked, plus I couldn’t really […] normally so I went out of my house to actually get stuff, and I can’t […] now because of it, the whole thing was pretty hard now that I think about it.

EC: Yeah. A common theme I keep hearing is “I was doing well, but then the pandemic happened and I couldn’t occupy myself in the ways I used to and that put me mentally in a hard place, living with my thoughts, things take over --”

003: Yeah for sure. I haven't been doing Xanax at all lately. It was just kind of in April and May that it was a daily thing. My […] at the time, was almost 6 years clean and relapsed and started doing Xanax because it was just so depressing just sitting in my apartment being stuck here.

EC: Okay we’re already at 27 minutes and I want to respect your time so if you have to go feel free. If not, I'd like to know what you think needs to change? What do you think of the province offering a safe supply of opioids?

003: It’s crucial. Absolutely crucial. Needs to happen. It’s so important.

EC: Do you think your situation could have turned out differently and you could have avoided the trauma you faced in […] if you did have a safe supply of opioids?

003: No, I have other addiction problems, not just opioids. I would have had to go there anyway, and the whole thing would have happened anyway, but I’m sure a lot of my friends would still be here if we had a safe supply. I've lost so many friends due to this stuff it’s just insane.

EC: That’s heartbreaking. I’m sorry to hear about your friends. If you could give advice to people in power, politicians or police, what would you say?

003: I don’t know, it’s a health issue. We need to stop being treated as criminals. A lot of other less nice things but that’s basically the just of it.

EC: Okay, so I just know that I’m going to be writing out this interview later and wondering why I didn’t ask things. Is there anything you’d like us to know about your experiences, or specifically at the rehab center, or anything you’d like to see us do to try and help?

003: It just sucks that there isn’t more available for low income people other than to go somewhere and have something like this happen to you. It really sucks. The only other places are in […] and no one can afford to go to somewhere that’s that much money. It sucks that there’s nothing else available.

EC: And what was available really hurt you…

003: Yeah.

*“[The exemptions] simplify my life a billion times from an administrative and logistical standpoint, but how we can use a triplicate [prescription pad] versus a telephone to do a prescription now… that is not the barrier to safe supply at all”* – HCP, addiction medicine physician.

EC: I just want to be mindful of time, we’re at 24 minutes. How has your practice changed during the pandemic? Has your prescribing changed, did you benefit from the federal prescribing exemptions? How did things look different for you and your patients?

004: What’s helped me during COVID is that a lot of the stuff that I have been talking about in my peer group, the things I wish I was doing before COVID, which I was trying to make happen in other places. […] is worth an entirely other discussion because there’s a whole story to that practice that’s really challenging, I’ll say. The beauty of being a physician and practicing independently and the reason why you can take that type of power to be very careful with it and be mindful of it, as long as we’re doing what we’re doing because it’s based on evidence we can kind of do what we want. Changes I was already making in my personal practice […], like writing people longer prescriptions, seeing them every few months instead of every month, I would write for 2-3 months. I was being more liberal with suboxone carries, things I could do in my own personal practice. But at […] I couldn’t do it unless I had massive support, if ever I went against the grain I would get my wrist slapped for it and it would cause a lot of problems. I’d have to very much follow exactly the guidelines at […]. Even though they serve the most vulnerable population in the city, a lot of their policies are very prohibitive and often the voice of physicians at […] is very muted and not respected. When we try to push envelopes on some things we get a lot of pushback. There’s no […] there and there really needs to be, because a lot of these are […] decisions. COVID helped me because I was like oh great, I can continue to do what I’ve been doing in my […], only I can now do it in […], isn’t that awesome. Another thing that changed for me was I could give some people methadone carries, and some I still couldn’t because I was really worried about their safety, but for some I could and that was really great. The interesting thing is that there’s a lot of attention about the verbal prescribing we do to pharmacies now, and it’s like, that makes my life easier from a logistical standpoint. It is so much easier that I can fax methadone prescription from my computer, and people can do pharmacy to pharmacy transfers without me having to get involved with that. That simplifies my life a billion times from an administrative and logistical piece. It changes nothing - it keeps getting brought up in these safe supply discussions, that they’ve changed this and that and pharmacies can now take verbal orders and therefore safe supply should now be easier. Absolutely does nothing to change the conversation about safe supply. How we use a triplicate versus a telephone to do a prescription… that is not the barrier to safe supply at all. The changes during COVID have made my life a lot easier. I think that I have had to make some uneasy decisions and I’ve worried about some people, and some of these things I have had to take back, sometimes that’s happened such as after the progression of carries, but for the most part it was reinforcing what I was doing in my […] and stuff I was trying to advocate for beforehand.

*“It’s almost impossible to get a hold of a doctor, or get an intake, or get in to see anybody. So, you’re dealing with being over the phone, all that fun stuff. I find it extremely frustrating, even now with my surgery, to talk to my own doctor has been a nightmare. I find basically we’ve just been ignored.”* – PWUD, 35-year-old.

EC: Yeah, okay. So now we’re going into stage 2. The question is, can you tell me what it’s like to use substances right now, during the pandemic. In your case, that includes what it’s like to get methadone, or anything on the street, whatever.

006: First and foremost, it’s almost impossible to get a hold of a doctor, or get an intake, or get in to see anybody. So, you’re dealing with over the phone, all that fun stuff. I find it extremely frustrating, even now with my surgery, to talk to my own doctor has been a nightmare. I find basically we’ve just been ignored. All of our efforts have fell by the wayside, like with the safe injection site, when if anything they’re needed more now because there’s less resources. I think there should be more resources right now instead of less because of the COVID. You’re not able to get a hold of people or access the resources you once could. Mental health has gone downhill. If you’re just starting out, you don’t see addicts walking about with masks and gloves and they’re the ones who are sharing equipment. There’s not enough harm reduction offered to these people. They’re not being educated enough. COVID has affected the whole mental health and addictions fight altogether and addicts’ lives matter it’s just they don’t care. The guy in the office has to wear a mask. They’ve closed shelters, which puts them directly into harm’s way. The funding for COVID was abused by many, yes, however those were the people that needed it. Now they’re being penalized. Life has become hard for everybody but definitely for the people who are on the streets. People didn’t wanna talk to a homeless guy on the street before, could ya imagine now?

EC: So, what do you think could have really helped during the pandemic that was missing? You’ve touched on how shelters closing didn’t help, the Halifix closing didn’t help. What else do you think was missing?

006: Like I said education would have been a big piece to it. Having people from that lifestyle doing peer walks handing out masks and educating other addicts on the things about COVID because right now I don’t think they even know what it is. They think it’s an old people disease or something that would never touch them. The education hasn’t been there and I think they should have been more involved in A) helping keep their own people safe and B) the government should have stopped shutting down shelters and forcing them into small places where there’s more chance of contracting these diseases. They totally have been forgotten. It has been made into one media thing for the middle and upper class man. The poor man is not being advocated for in any way. You asked about my income, the small job I had doing […] just got cancelled. I wasn’t able to get a hold of the doctor when I had an abscess in my leg. I could have died of a blood infection. They don’t want anything to do with you. I don’t know if they feel “oh it’s all their fault” or what their deal is but I had a lady in the surgery who poked herself in my OR and they must have taken 10 bottles of blood from me [to screen for blood borne infectious diseases], treated me like I was a leper, and you know…

EC: Oh no…

006: I definitely feel we were dehumanized and as a last resort of people to educate. I know they’re handing out COVID cheques to these people and I know they’re being overused but what about handing out a small honorarium to these people to attend a course where they get taught. Or here, here’s a box of masks and for every person you get to and answer certain questions you get 25 cents or something, instead of picking up bottles? That’s just off the top of my head, but there’s many different things they could have done to include a lower standard of people. Their only concern has been the upper and middle class people. Closing down shelters especially, where are these people supposed to go?

*“I went without antibiotics for over a week until my leg was to the point of seeping, abscessing… I could have died of a blood infection.”* – PWUD, 35-year-old.

EC: So, it sounds like you couldn’t get a hold of your doctor, well did you hear the Federal government made some exceptions that were supposed to help prescribers be more lenient when it came to prescribing drugs during the pandemic, especially controlled substances.

006: Really, I wasn’t aware of that…

EC: So basically, they can prescribe over the phone, which they couldn’t do before, and they can transfer prescriptions between pharmacies. Did you know of this and did you benefit from it?

006: I wasn’t told about it but I was offered the opportunity because, like I said I’m staying with […] for a few days and then I’m going to be transferred to […] and they called me to offer to transfer [my Rx] there and offered delivery. So, although I wasn’t told that it was an option, I was told that I could. So, it was offered and that was fine. And the phone thing, yes, but you have to get them on the phone first. Like I said, I went without antibiotics for over a week until my leg was to the point of seeping, abscessing, could have very well went to sepsis or I could have died of a blood infection.

EC: How did you get that treated in the end?

006: I finally, after almost a week, the doctor called, they sent […]. […] showed up with […], with the […] sign, and I’m sorry -it is embarrassing. And […] was like “wow, that’s bad”. […] was the one who prescribed me Keflex, the most strong antibiotic. I told […], I’m going to surgery on Monday and if I have any infection in my body they’re not going to operate on me. […] was really good at making sure I got what I needed there but if it weren’t for that… […] never called me back. I understand […] is overworked, but that’s what I mean, why are these doctors so overworked? Why aren’t there more doctors in this field of mental health and addiction? They’re actually taking them away.

EC: Exactly. It’s not right how overworked […] is and you suffer because of it.

006: I believe it but frig.

EC: It’s not fair that that falls on you.

006: I just heard my parents say in the background, it seems the government does not care when it comes to the lower class of society.

*“The greatest challenge has been the disconnect... Because that’s the crux of harm reduction, of the work, is that connection. Meeting people wherever they’re at in their lives. That contact. That engagement. That dialogue. That respect. That care, compassion, respect and dignity that is so integral. So that has been very challenging for people. And for us, doing our work. We miss the people. We miss that. So [switching to telemedicine] has been a challenge.”* – HCP, program lead.

EC: It seems to me through the interviews that one of the barriers that keeps coming up is how it really seems that the general public, even maybe policy makers, don’t understand addiction at all. That’s been pretty troubling over the course of this project. Okay, if it’s alright with you, I’d like to get into how the pandemic has affected your work?

013: Yeah, I mean I think the greatest challenge has been the disconnect. The connection. Because that’s the crux of harm reduction, is that connection. Meeting people wherever they’re at in their lives. That contact. That engagement. That dialogue. That respect. That care, respect and dignity that is so integral. So that has been very challenging for people. And for us, doing out work. We miss the people. We miss that. So that has been a challenge. One of the benefits of the pandemic has been that opiate treatment historically has been very stringent. I think it’s opened our eyes that we can be more civil and less stringent in allowing people to have more autonomy with how they participate in their treatment, in gaining carries and those kinds of things that we’ve had to manage the risk of the pandemic and risk of overdose. In there, there’s been this realization that wow, we haven’t had any disasters as a result of this pandemic and there’s been an influx of carries. So that’s been a benefit. That has been good to see. I think the mental health of people has been a challenge. The isolation. The CERB. Wow, wouldn’t it be great if everybody was given that amount of money to live every month. But I think given the context of the way in which it was given that some people might have been at increased risk, because of the way in which they were getting the funding, and many were getting income assistance as well. There were some challenges around how that was being dispersed. Creating some risks for people and we’ve heard it from substances users.

EC: I’ve definitely heard that as well.

013: Yeah, I think that 2000 a month is by far fairer than the ,5 6, or 700 they get per month. I’m not debating that. I guess people’s opportunities as to what they’d do with those funds were limited. The other thing, I know there was safe supply provided for folks. I know there were some people doing well with that, and I’ve heard of other folks doing well on one modality of treatment and then they destabilized because of the safe supply. So, you know, it’s sort of, again, in this work how do you balance the risks?

EC: If there’s anything I’ve learned is that it’s so complicated and different for everyone. It’s hard to make sense of it.

013: And then there was concern about the safe supply maybe being cut off. Concerns around that we heard from people.

EC: So besides what you mentioned, a little more leniency and carries were distributed. In what other ways did […] respond to the challenges that the pandemic…

013: Oh my goodness, we were giving gift cards to people. Increasing our calls with folks. Obviously we couldn’t have programming and such. Obviously the […] were busy on the telephone checking in with people and the physicians. A lot of telephone appointments.

EC: It’s really good to hear that there were no big disasters, like you said. Okay the next question is, would a safe supply of drugs benefit or harm your patients and like you said it depends..

*“We did feel really worried about that (spreading COVID-19). So, we tried to minimize a lot our contacts with people, but we still found it was a really delicate balance because we thought in some ways the risk was worth it to be out and about because COVID isn’t the only cause of death.”* – HCP, mobile health service.

EC: I do want to get at how these challenges have been impacted by COVID? Are there peer support workers that are still able to, is everything still on hold, how did you –

016: Yeah, so, COVID affected this group disproportionately because they rely on free resources and services to live. When COVID happened, everything shut down. I’m talking everything. They couldn’t even, there was no space for them to sit during the day, except for outside. Even then, people kick you off the side walk or whatever. The libraries were closed down, they weren’t allowed indoors. And then all of the support people were not working in person. They were doing things via phone or not at all. These folks don’t have phones, or don’t have the ability – I have many patients who can’t use a phone. Or get so frustrated, their anger management is not good enough to deal with the complexity of a phone. Some of my patients. So that was wiped out. And then just the pure fear that was happening for everyone, including myself, including everyone. The unknown and the fear in the moment. Everything’s calmed down now, but if you think back we didn’t know was going to happen, if we were going to be the next Italy. So, everyone’s preparing, there’s lots of anxiety. If you have someone who already has insane high anxiety, and that’s put on top, I mean. We’ve had lots of suicides. Lots of relapses. It made it interestingly, I don’t know, I’m speaking a bit out of turn because I’m not a drug user and I wasn’t in the scene, but I’ve heard that it was really hard to be a drug dealer, or get drugs to people, because there was no one on the streets. No one was around. So, people who are doing the busy work of delivering illicit drugs, it was really highlighted or obvious, and way easier to keep track, because they kind of rely on anonymous, trying to slink in to looking normal. So, it was really eerily, or if you remember in the first few weeks, but it was like there was no one on the road. It was a ghost town. It was really obvious. And then so much more. I don’t know if I really answered your question but –

EC: You did, definitely. I’m curious for you, were you still able to go around and practice?

016: Yeah, we were really aware that we could be quite a vortex. Do you know what I mean by that. It was just basically […] and I that continued to, and […] a little bit, but […] and I decided we’re still coming into work. We have to see bodies. The shelters were really anxious, the people were really anxious, so we found if we just visited, our physical presence just brought some calm. And, […] and the other RN talked a lot about how we were very worried because we see a lot of people and from going to a lot of different places, if we unknowingly came into contact with someone with COVID, and all of a sudden we have transferred it to a lot of people. We did feel really worried about that. So, we tried to minimize a lot our contacts with people, but we still found it was a really delicate balance because we though in some ways the risk was worth it to be out and about because COVID isn’t the only cause of death. And everything else is kind of sky rocketing. People were monitoring their heart meds, insulin, all kinds of things, not to mention mental health. And that’s still happening right now. So, we just put on masks, at one point we were gown and gloving, and we just tried to go to agencies and stand outside, or have people come outside to us and stand really far back. We didn’t let anyone in the […] for a while because we were worried that would be quite the vortex. […] and I were monitoring the news like crazy trying to grasp for any information we could because then it’s out care, but we just made ourselves available but I think the best work we did then was supporting agency staff. Because they, of the shelters and everything, because they were so stressed. And they were the means to providing basic care for so many of our clients. So, if we could keep them calm then they could provide more care for more people. So, we did focus on staff a lot and that seemed to be good. And of course, we were heavily involved in lots of decision making, advocating, meetings, because of course in […], you need 18 meetings to make one choice and that’s really frustrating. Anyway, luckily we were actually mostly asked to come to the table which is huge. We also have to acknowledge the progress that has happened in our society and health authority, to see that they called us and they asked for our opinion. That means they acknowledged that the work we do is important and influential and we have some information. And that is different, that has not happened before. That is really positive.

EC: Yeah, that’s awesome. But also, unfortunate that that wasn’t happening before.

*“Mainline for example, they are handing out harm reduction things. And during the pandemic they started giving a lot of pharmacies essentially little gift bags of drug use equipment that you would need…which has been really helpful.”* – PWUD, 26-year-old.

EC: From what I’ve been hearing you’re definitely not alone in that. So, would you say that you feel stigmatized as someone who uses substances? And maybe specific to the pandemic, but also just in general, by say, healthcare providers? Or the general public, or family members?

002: Yeah. Absolutely. If you were to look at me, you’d never think I look like a drug addict. Even though, like, there’s no look of someone who’s a drug addict. Anybody can look like an addict. But, in the healthcare system, I get treated so differently and people can be very hostile towards me. I almost feel like I’m being interrogated. For example, in November I ended up getting a really really bad [health problem] and I had to go to the hospital for four days. I had to tell them “Hey, just so you know, I’m on methadone so I'm going to need that while I’m here”. I think because of that I got treated differently by the nurses and I would have people come in and essentially check in on me to essentially see if I wasn’t doing any other drugs or anything else. You know, if you tell people that you’ve dealt with these things, outside of the healthcare system, I’ve had so many people say horrible things about me just because, I would be, I remember I had [friends]. We were all friends and everything was fine but I was secretly hiding that I was an IV opiate user and I used every day. They found out because they ended up snooping through my room which was very weird and they ended up […] and started all these rumors about me that weren't true. I was like “I was friends with you for months and we were all good and now suddenly because you found out I do drugs, you’re gonna […] and spread all these horrible rumors about me that are entirely baseless?”. Even one of them was a [helping profession], and I was like, this is ridiculous. And those are only two examples of ways that I feel people have treated me differently.

EC: I’m sorry to hear about that. It’s awful but also seems so common. I’ve been hearing about people who chose these professions to help people, but are still kind of perpetuating this stigma.

002: Exactly.

EC: So, you mentioned that your supply of drugs is definitely not stable during the pandemic, hydromorphone has dwindled or gone up in price, so now you’re using heroin since it’s more accessible. Could you walk me through what using looks like to you. From acquiring it to finding a space to use. And has this changed a lot compared to before the pandemic?

002: Not really, nothing has really changed in terms of getting it, acquiring it, and doing all of the using parts, except for the part where the drugs in Halifax are changing. But, […] for example, they are handing out harm reduction things. And during the pandemic they started giving a lot of pharmacies essentially little gift bags of drug use things that you would need. It has been more convenient to get the stuff you need compared to before the pandemic which has been really helpful. But nothing has changed much in terms of getting it and doing it. I do know that heroin is a lot more dangerous, there's fentanyl involved with it, and I experienced, for the second time, I overdosed. And I had a friend who overdosed. So that’s something that’s changed during the pandemic because I wasn’t used to people overdosing because it’s hard with opiates but even a little bit of heroin, because you don’t know what’s in it, it’s so easy for people to overdose. That’s something I’ve noticed has gone up and have experienced seeing and have had to revive people, which has been really scary. That’s really the only different thing that I can think of.

EC: When you’re actually acquiring these substances, were you concerned about leaving your house at times when you were told to stay home? Did you feel you were put at higher risk having to leave and get these substances that you really need?

002: Um, no. There’s nothing like that. We were basically free to come and go as we pleased. My [relative] lives [in the house] and they didn’t question us about where we were going and what we were doing.  Not really no.

EC: I’m not sure if you’ve heard but the Federal government made some exceptions for prescribers. Basically, they made it so they can be a bit more lenient with prescribing, like prescribing over the phone, or with carries. Have you benefited from any of these exemptions?

002: Yeah. I go to [OAT clinic] to get methadone and have been doing over the phone with doctors to get refills which have been, honestly, super super convenient. And with my doctor who I’m working with to taper off the Xanax, I talked to [them] over the phone and I was open and honest with [them] about everything and that went well. I find, for me, the experiences that I’ve had, which has only been very few, that it’s been very beneficial for me because then I don’t have to go out. It’s kind of easier talking to people over the phone than face to face. Because even if I went to my doctor's office and tried to explain, because when I talked to my doctor about getting off Xanax, if I was there in person, I would have had a much more difficult time. But being over the phone I was able to talk more openly because I wasn’t there in person. If that makes sense? Social anxiety makes it hard to explain things sometimes.

EC: That definitely makes sense. I find it easier to chat over the phone instead of video call for example.

002: Yeah, exactly.

*“My dream is that eventually we’ll have a fully resourced, professional consultation service at the [local hospital] and be able to provide that [care] around the clock”.* – HCP, resident physician.

EC: Okay, awesome. Can you tell me a little bit about the on call addiction medicine group that you helped put together?

018: Yeah. At the […] there aren’t any formal addiction supports for people with addiction, so patients who use drugs or alcohol when they are admitted to hospital. There isn’t really very much training amongst the staff that work there. So, over the past few years a few residents who are based out of the hospitals have sought out extra training in addiction medicine and partnered with community based addiction physicians who don’t have hospital privileges. We’ve developed a group where consultations can come into our group and residents will see them in hospital and do an assessment while supervised remotely from one of the community based physicians. And then usually the standard is that they will come into the hospital and see the patient, face to face at least once, and then we will be able to transition care, usually to them [the community based physician] or a related colleague after discharge. We’re primarily seeing people with OUD or people who inject drugs, often semi urgently in the context of opioid withdrawal or ongoing use or addiction in hospital. We’ve also been involved in a few cases with complex alcohol use withdrawal or use disorder.

EC: What was the impetus to get this going? You mentioned there wasn’t a lot of training already among the staff at the hospital?

018: Yeah. So, we very regularly --patients who have severe SUD or who inject drugs are admitted to hospital with medical complications that are quite complex and require multidisciplinary or multispecialty team based care, might be there for surgery or heart valves or spinal abscesses or long term antibiotics. The prior standard of care, when I was going through medical school, was that we would do all of these things for care but completely ignore the underlying SUD that caused the problem or forced people into withdrawal so they left hospital AMA. In the community I had seen the local harm reduction organizations and their approach to care, where they’re trying to meet people where they are and figure out what they need and support them holistically, and not judgementally and with lots of love. I realized that was completely missing from the hospital. I was involved in a few patient cases in med school where we clearly did a disservice to that patient and caused a lot of harm by not treating addiction and not treating pain appropriately. And all the health care providers didn’t know what to do and were scared of making things worse, of afraid of doing the wrong thing. I first talked to […] about it because he is a community addiction doc who would come in every once in a while if they wanted to start somebody on methadone on a Friday afternoon, a couple times a month, after his clinic. [They] was agreeing that we needed a lot of better supports for people in hospital. [They] was kind of saying who is going to do it, and if it’s going to be you then what training do you have, kind of thing. So, I started seeking out the extra training and talking and talking to other residents about it. I think a lot of others felt the same moral distress, nobody knew how to help clearly something was wrong. Clearly we weren’t helping. And it’s kind of snowballed from there.

EC: Wow. As someone who has no clue what goes on inside a hospital it is kind of shocking to me that so recently this didn’t exist at all. I’m wondering, do you get paid to do this or is it completely volunteer?

018: No, we get a salary as residents though. We don’t get paid any extra. I think most of the docs we work with don’t even bill for their involvement on the consultations for us. So, my dream is that hopefully eventually we’ll have a fully resourced, professional consultation service at the QEII and be able to provide that around the clock. One huge issue we have is that the supervising physicians are never available to come into the hospital during the day time because they have their own clinics. And residents are only doing it on the side of the desk post call or if a weekday clinic ends early or in between consults. It’s really patchwork that way. We can’t really keep up. But yeah it is pretty astounding whenever we tell people that there’s this need everybody is like “Oh yeah why haven’t we been doing this? Why don’t we have these skills?”. The answers are pretty complicated and have to do with the history of addiction care and how it wasn’t science-based for a long time and then it was because drugs are illegal, so care for OUD and stimulant use disorders were locked off with the rest of medicine and stigmatized and misunderstood so it’s kind of developed separately. And now I hope and think it’s being reembraced by mainstream medicine. But when I was in med school and still, there’s no one on staff at the hospital who had a methadone prescribing exemption for addiction. The palliative care docs could do it for chronic pain but there was no one who was comfortable or trained to start methadone in the hospital. Which is insane when you need to be there for sometimes 8 weeks for IV antibiotics and surgery, obviously that’s not okay to not offer people the standard of care.

EC: Yeah. Wow. I honestly had heard a little bit about this but didn’t realize just how lacking the services were in hospital.

[…]

EC: I’m curious, has anything to do with the service or this group in general, how do you think COVID has affected any of this service delivery?

018: Yeah, it’s a bit complicated because we only see just a small snapshot of the big picture in terms of whose being admitting with medical complications or overdose. But I’d say it’s affected it in a few ways. One is that, I’ll just think in the different ways I’ve seen doing these consultations,  so a couple have been that they haven’t actually seen their prescriber in the community and have only had their things adjusted by phone. Some people have been afraid to go into clinics to get started on treatment because of fear of COVID, like going to the pharmacy. A lot of people don’t really know that a lot of stuff has been moved over to phone-based where they don’t need to be in person that much but are still concerned about having to pick up at the pharmacy every day. We’re seeing a lot of people in hospital, we’re seeing that overall the numbers have gone up and we’re not able to, maybe it’s confounded by the fact that many people know about the service, but we’re getting many consults a week now. Whereas we used to get just 1-2 per week. So, part of that is why I’m seeing folks, they’re talking about being isolated,  using more, because they don’t really have anything else to do, or any distraction, they fell cut off, don’t have as much support, so they’re using more and using more dangerously, or more chaotically.

EC: That’s definitely been a common theme from the folks I’ve spoken to.

*“A lot of these women are escaping abusive relationships and drug addicts and for them to have to go down there and face that, I didn’t think it was an ideal spot [the new location]… This is coming from a woman that was involved in prostitution and abusive relationships.”* – PWUD, 35-year-old.

006: My point was, as a female, a lot of females don’t want to go down there [to the OPS]. Their ex-boyfriends are down there [at a neighbouring men’s shelter], that beat them. So as a woman I know I wouldn’t feel quite comfortable going down there with my drugs. A lot of these women are escaping these abusive relationships and drug addicts and for them to have to go down there and face that, I didn’t think it was an ideal spot.

EC: That’s a really good point.

006: I voiced my opinion that I wasn’t included [in the decision to put the OPS in that location]. This is coming from a woman that was involved in prostitution and abusive relationships. I find the first hand voice, as much as they act as if it’s so important and they’re listening, at the end of the day it doesn’t make a difference. They try to make it seem like it does, i don’t know if that’s for their supporters or their grants, or what but we’re all starting to feel a little frustrated that it’s all been one big scam.

*“When you can’t pay more than $600 per month in rent, you’re going to have thousands of roommates in the form of rodents and insects and bedbugs and all that. And that’s just the way it is. You have to learn to live with those rodents and insects”.* – HCP, mobile health service.

EC: I never really thought of that. Do you see that […] is steering in any new directions? Or doing the same thing just at a bigger scale?

016:   That’s a really complicated and intricate question. I think the main lifeblood of […] is identical to when it started. It’s providing authentic love and compassionate care to people in the moment when they need it. And trying to help navigate people in a really kind way through this horribly aggressive and awful healthcare system that we have at times –it’s really traumatic. That is absolutely the same, there’s no question. The way we do it, and are allowed to do it, and have to do it, and all of those things, is a little bit different. In that before, and also it’s just so complicated because the world is just so different, even in the last five years. The intensity on the streets, and since COVID, oh my god, the intensity the anger and anxiety is just through the roof in people. That makes things really different too. The housing crisis here, insane. There’s just no where to house people. I just made a comment the other day to somebody and I caught myself saying out loud, and I just was floored, I said, “I’m really sorry. But when you can’t pay more than $600 per month in rent, you’re going to have thousands of roommates in the form of rodents and insects and bedbugs and all that”. And that’s just the way it is. You have to learn to live with those rodents and insects. Like that’s fucking crazy that I caught myself… I’m usually someone who just will not accept inadequate humanity. I don’t know how to say that. But I have to now. Because it is better for them to be in a house with a bunch of bedbugs and cockroaches than completely homeless with no shelter beds available and the weather roaring. We’ve banged on every door and tried to advocate and strategize and collaborate and, to no end. It’s a work in progress, we haven’t given up, it’s still happening as we speak. But it’s so inadequate right now.

EC: I don’t mean to derail us, but I’m just so curious to what you think are some potential ways for, not necessarily solutions because I know it’s not that easy, but how can we address this housing crisis? It keeps coming up in all these interviews.

016: This is probably too utopic but I have fantasies all the time of winning the lottery and buying two or three huge buildings and providing people with nice durable clean apartments for $535 which is what they get from income assistance. And also staffing them with cleaning, home care, social work, lots of man powered resources in those buildings. It would be important to have three different buildings because you always have lots of conflict between different people who are required by law not to be around each other, so you can’t have just one big thing. But basically, also it’s as simple as providing more affordable housing units. It’s that simple. You can’t get a one bedroom or bachelor apartment that isn’t deplorable for under $800 right now. It’s just insane. That sounds really simple but in face in our current culture it’s really complicated unless you have a bunch of money and are feeling philanthropic. It would be fantastic, and maybe if I had more time. See that’s the other problem, I’m extremely busy. I can’t describe. My voicemail, I’ll leave the phone for two hours and come back and there’s 49 missed calls and 60 texts. You know it’s nuts. I’m also just so busy with requests from pharmacy and filling prescriptions. My head and my body is totally consumed by in the moment tasks so I don’t have enough time to do some of the strategizing.

*“The street supply of drugs, or prescription supply of drugs really dried up. Halifax has historically been a prescription opioid town. If somebody has opioid use disorder in NS, they are historically addicted to prescription drugs. What’s happening is because the doctors weren’t able to provide care or prescribers were providing really acute emergency care, the street supply of prescription drugs really dried up. That was our way of having a safe supply.”* – HCP, Pharmacist.

EC: So once the pandemic hit, how did operations change?

005: Blagh. The NS government, and I will go on record to say this, let pharmacists down. They let community pharmacists down. What happened earlier in the pandemic, I picked up a few shifts in the community and it was absolute pandemonium. A mess. PPE wasn’t provided. None of this plexiglass stuff. People were panicking and trying to fill their medications way too early. It was absolute pandemonium. I’ve been a pharmacist for a long time and it was some of the worst shifts I’ve ever worked. On that note, pharmacists were hung out to try and left to pick up the pieces with a lot of family doctors clinics shut down early on and said we can’t do it. The telehealth stuff hadn't been stood up yet so there was just this gray area where pharmacists were meant to pick up the pieces and take on a much bigger role than they historically have ever had to. But also, what happened early on is places like […] were forced to shut down by public health because they didn’t have the PPE and all that stuff in place so they just had to close down. People did not have as easy access to these brown bags and safe needles and gear as they normally would. They were able to get back open fairly quickly. That puts people at risk. The third thing that puts people at risk, the major thing, is that street supply of drugs, or prescription supply of drugs really dried up. NS has historically been a prescription opioid town. If somebody has an OUD in NS they are historically addicted to prescription drugs. In Halifax in the valley it’s always been hydromorphone or Dilaudid, and in CB it’s been Oxycontin or Oxycodone. That’s still pretty much the case except what’s happening now with the advent of 3D printers, people are able to print tablets that look exactly like the Dilaudid that people are used to buying on the street but they’re full of fentanyl and Draino. What's happening is because the doctors weren’t able to provide care or, the prescribers, were providing really acute care emergency care, the street supply of Rx drugs really dried up. That was our way of having a safe supply. Yes, it was a safe supply, but it was safe because it was pharmaceutical grade stuff. If it says 8mg on it and it was in a Rx bottle, you’re at a higher likelihood of actually injecting 8mg of hydromorphone. But as I say with the advent of 3D printers people can make it look the same and it may not be, but you’re more likely to have a safe supply with that. What happened then is if you have fewer prescription tablets on the street the illicit market takes over. The safe supply became increasingly tainted and we saw this trend of increased overdoses. I had a pharmacist reach out to me in […] that people were buying what they thought was opioids but it ended up being de-wormer for dogs and there was fentanyl mixed in it. So, people were overdosing on fentanyl because they were used to injecting hydromorphone, so 100X that is what they’re taking plus de-wormer which is a neurotoxin. So, they were overdosing and also having major seizures which is not a part of opioid overdoses. That’s all because the supply in NS and across the country because the borders were shut down, the illegal market of shipping stuff into Canada was curtailed. The supply became increasingly tainted and dangerous. People didn’t have as easy access to safe needles and gear, and didn’t have as easy access to a safe supply. This put people at incredibly increased risk and we did see, I work with […], and she did see an increase in overdoses and I believe there was an overdose during that period of time. I think we can contribute to an increasingly tainted supply because of the COVID-19 pandemic.

*“[People are] straight up pulling out $2000 and buying everything that they can because they can at the time. Not that I’ve done that specifically but there’s been a lot of panic buying for people and their supply. People that usually have sold their prescriptions no longer do because they’re so afraid of running out. So, it’s just leading to this panic.”* – PWUD, 21-year-old.

EC: Yeah, okay thank you for sharing that. This will change pace a little bit, but I’m wondering if you could walk me through what using looks like for you right now. From acquiring it to using. It was hydromorphone did you say?

001: Yeah and fentanyl.

EC: What does that look like for you?

001: Currently, at the moment, I’m not doing either of those things. But over the [early] pandemic I was. It’s only been recently. For a while it was once […] and everything stopped I started to have a lot more free time, and as lock downs started to go into place, and CERB money and all that, I started to find myself having more and more time and less and less purpose or meaning or things to do in a day. So, I started to increasingly do more opioids, especially considering previously I had been very solid for 6-7 months doing well. It started out mostly just hydromorphone but as the pandemic went on it became more and more fentanyl as the supply of hydromorphone very quickly vanished. What do you mean by how does it look finding it?

EC: As I’m asking this question I realize you probably don’t want to tell me how you acquire these drugs.

001: I mean, you never know where they’re coming from. It’s always up in the air. One day you might get a text from one person. Another day you might get a text from another. You never know where it’s coming from and people are panic buying everything they can. Straight up pulling out $2000 and buying everything that they can because they can at the time. Not that I’ve done that specifically but there’s been a lot of panic buying for people and their supply. People that usually have sold their prescriptions no longer do because they’re so afraid of running out. So, it’s just leading to this panic. For the most part I’ve really stuck to staying on my suboxone because it’s near impossible to get a consistent supply. That’s definitely a very large risk: that you never… one day it’s out front of [local shelter] and the next day you could be going to [different neighborhood] to meet someone, it really changes all the time.

EC: The next question is “is your supply of drugs stable” I guess you just answered that. No, it doesn’t sound like it’s very stable at all.

001: It was before the pandemic. Before the pandemic, if I wanted, I could literally just call somebody. I could catch a bus, be there in 15 minutes, but now you pretty much… if you don’t have someone on dial that you can call, you have to roam around [street name] for hours until you see somebody who might. And it’s depending on if you know them, they may price gouge. It’s certainly very rough at the moment. I feel bad for people who don’t even have Suboxone or methadone because holy shit… yeah.

EC:  So during the peak of social distancing, would you say most days you’d have to leave your house and go and try to …

001: Yeah. I honestly felt very shitty about that. I wish that, at the time, I could have just gone to my doctor. That’s all it would have took. A note on a pad. And I’m not saying it’s a perfect thing. But if it’s between that and disparity and exposure and risk.. Like going on the bus peak pandemic because that’s the only way you’re going to get something. That is a very dangerous game to play. And coupled with the fact that if you get sick from COVID and you get sick, it’s not gonna be good. So yeah, I definitely repeatedly exposed myself to unnecessary risks during the Covid-19 pandemic.

*“People are being forced into a situation so desperate that they’re going to that purple fentanyl of unknown purity and source. It could even not be fentanyl it could be carfentanil and that really sucks about this pandemic, everybody’s got money and everybody’s looking, everybody’s getting money around the same time and it’s just a free for all. I have both witnessed and heard of overdoses that have occurred because of it.”* – PWUD, 21-year-old.

EC: I agree. Thank you so much again for doing this interview. We’ll move on to the next series of questions. Can you tell me a little bit about what it’s like to use substances right now during the pandemic?

001: There is a lot of disparity. People are paying CERB/EI money and the drugs are so expensive and people blow through all their money in no time. For example, 5mg of hydromorphone is going anywhere from 15-30 dollars. That is insane. Pre-COVID it was 7-10 dollars for 5mg. As of the past couple months I’ve been hearing more and more about fentanyl trickling into the streets, I’d imagine because of the reduced supply of opioids that is going on. It’s kind of forcing people to go to a very tainted supply.

EC: Alright, and when you say you’ve heard about it, is it people you know? Or you’re hearing of people's experiences that they’ve been using fentanyl without knowing?

001: I’ve talked to multiple people who have stated that they can get it for me. I’ve had previous experience with this purple fentanyl that likes to make its way out to Alberta and BC and I’ve had some experiences with that. And one particular thing that really scares me is that I’ve actually seen it here in town and that really sucks because before we had people using, well it's not great that people are using hydromorphone, I don’t like that idea, but now people are being forced into a situation so desperate that they’re going to that purple fentanyl of unknown purity and source. It could even not be fentanyl, it could be carfentanil and that really sucks about this pandemic, everybody’s got money and everybody’s looking, everybody’s getting money around the same time and it’s just a free for all. I have both witnessed, heard, and heard of overdoses that have occurred because of it.

*“I do know that heroin is a lot more dangerous, there’s the fentanyl involved with it, and I experienced, for the second time, I overdosed. And I had a friend who overdosed. So that’s something that’s changed during the pandemic because I wasn’t used to people overdosing… you don’t know what’s in it, it’s so easy for people to overdose.”* – PWUD, 26-year-old.

EC: You mentioned that your supply of drugs is definitely not stable during the pandemic, hydromorph has dwindled or gone up in price, so now you’re using heroin since it’s more accessible. Could you walk me through what using looks like to you. From acquiring it to finding a space to use. And has this changed a lot compared to before the pandemic?

002: Not really, nothing has really changed in terms of getting it, acquiring it, and doing all of the using parts, except for the part where the drugs in Halifax are changing. But, […] for example, they are handing out harm reduction things. And during the pandemic they started giving a lot of pharmacies essentially little gift bags of drug use things that you would need. It has been more convenient to get the stuff you need compared to before the pandemic which has been really helpful. But nothing has changed much in terms of getting it and doing it. I do know that heroin is a lot more dangerous, there's fentanyl involved with it, and I experienced, for the second time, I overdosed. And I had a friend who overdosed. So that’s something that’s changed during the pandemic because I wasn’t used to people overdosing because it’s hard with opiates but even a little bit of heroin, because you don’t know what’s in it, it’s so easy for people to overdose. That’s something I’ve noticed has gone up and have experienced seeing and have had to revive people, which has been really scary. That’s really the only different thing that I can think of.
