# Supplementary material for: ’More of the same, but worse than before’: A qualitative study of the challenges encountered by people who use drugs in Nova Scotia, Canada during COVID-19
Source: PLoS One. 2023 Apr 5;18(4):e0283979. doi: 10.1371/journal.pone.0283979 (PMC10075390; doi:10.1371/journal.pone.0283979)
Supplement: S2 File — (DOCX) [file pone.0283979.s003.docx]

**Interview Guide for PWUD**

Stage 1

- What is your name?
- How old are you?
- Where are you living?
- How long have you been in Halifax/Dartmouth?
- Have you ever been homeless? If so, for how long? Have you been homeless more than once?
- Do you use substances? If so, which substances do you use?
- Are you earning an income right now?
- If not, how are you making ends meet?

Stage 2

- Can you tell me about what it’s like to use substances right now?
- Do you feel stigmatized as someone who uses substances? If so, has that changed at all during this pandemic?
- Can you walk me through step by step what using looks like for you? From acquiring it, to finding a space to use?
- Is that [see previous question] different compared to before the pandemic? If so, how is it different?
- Is your supply of drugs stable?
- Have you benefitted at all from the recent Federal exemption that allows prescribers to be a bit more lenient when it comes to prescribing drugs during this pandemic?
- Are you using any services from local organizations during this pandemic? If so, which services/organizations?
- Can you think of anything (services, support, shelter, food, healthcare) that could have really helped you during this pandemic that was missing?

Stage 3

- Once the pandemic is over, what do you think you’ll need?
- If you could give advice to people in power, people who make decisions like police, what would you say to them?
- Has there been any changes during this pandemic that you would like to see continue even after the pandemic is over?
- What sort of changes would you like to see after this pandemic is over?

**Interview Guide for Healthcare Providers**

Stage 1

- What is your name?
- Where do you work?
- What is your position at work? Can you tell me a little bit about your role?
- How long have you been working in this position?
- Do you work with people who use drugs? If so, around what percent of your clients/patients use drugs?
- Do you work with people who are experiencing homelessness? If so, around what percent of your clients/patients experience homelessness?

Stage 2

- What sort of services do you offer to people who use drugs?
- Can you tell me about how your job has changed during this pandemic?
- What challenges has the pandemic introduced into your work?
- Are your patients/clients able to access the supports/care/treatment they need during this pandemic?
- How would a safe supply of drugs would benefit or harm your clients/patients?
- Which services have you offered that have helped your patients/clients during this pandemic?
- Have your practices changed since the recent Federal exemption that affects prescribing practices during the pandemic?

Stage 3

- How has the government supported you during this pandemic?
- How has your employer supported you during this pandemic?
- What supports were lacking to you as an employee during this pandemic?
- Has there been any changes in your work/practice that could/should persist well after this pandemic?
- How do you think your work will continue after the pandemic is over?
- What services/organizational practices failed during this pandemic?
- If you could create a new law or change an existing one to better serve the population you work with, which law would you change or create and why?
